# Ultrastable Cu‐Based Dual‐Channel Heterowire for the Switchable Electro‐/Photocatalytic Reduction of CO_2_

**DOI:** 10.1002/advs.202302881

**Published:** 2023-07-02

**Authors:** Bo Li, Xiao Liu, Bin Lei, Haiqiang Luo, Xize Liu, Hengzhi Liu, Qinfen Gu, Jian‐Gong Ma, Peng Cheng

**Affiliations:** ^1^ Department of Chemistry Key Laboratory of Advanced Energy Material Chemistry (MOE) and Renewable Energy Conversion and Storage Center (RECAST) College of Chemistry Nankai University Tianjin 300071 P. R. China; ^2^ Australian Nuclear Science and Technology Organization (ANSTO) Melbourne, Australia, 800 Blackburn Rd Clayton VIC 3168 Australia

**Keywords:** CO_2_ reduction, Cu nanowires, electrocatalysis, metal–organic frameworks, Mott–Schottky

## Abstract

Catalytic conversion of CO_2_ into high value‐added chemicals using renewable energy is an attractive strategy for the management of CO_2_. However, achieving both efficiency and product selectivity remains a great challenge. Herein, a brand‐new family of 1D dual‐channel heterowires, Cu NWs@MOFs are constructed by coating metal–organic frameworks (MOFs) on Cu nanowires (Cu NWs) for electro‐/photocatalytic CO_2_ reductions, where Cu NWs act as an electron channel to directionally transmit electrons, and the MOF cover acts as a molecule/photon channel to control the products and/or undertake photoelectric conversion. Through changing the type of MOF cover, the 1D heterowire is switched between electrocatalyst and photocatalyst for the reduction of CO_2_ with excellent selectivity, adjustable products, and the highest stability among the Cu‐based CO_2_RR catalysts, which leads to heterometallic MOF covered 1D composite, and especially the first 1D/1D‐type Mott–Schottky heterojunction. Considering the diversity of MOF materials, the ultrastable heterowires offer a highly promising and feasible solution for CO_2_ reduction.

## Graphical abstract

1D dual‐channel heterowires, Cu NWs@MOFs, are constructed with switchable electro‐/photocatalytic mode, excellent efficiency and selectivity, adjustable products, and superior stability for the reduction of CO_2_, which leads to the first heterometallic metal–organic framework (MOF) covered 1D composite, and especially the first 1D/1D‐type Mott–Schottky heterojunction.

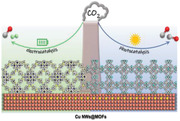

## 1 Introduction

Carbon dioxide (CO_2_), the major factor in global warming, is an abundant and inexpensive C1 building block to provide high value‐added chemicals and/or fuels.^[^ ^1^, ^2^ ^]^ Accordingly, efficient catalytic conversion of CO_2_ has great significance for the sustainable development of human society, green synthesis of fine chemicals and clean energy for carbon neutrality.^[^ ^3^, ^4^ ^]^ Among the diverse pathways, the electro‐/photocatalytic CO_2_ reduction is a promising method driven by renewable electricity sources (e.g., wind power, hydropower and nuclear power) and solar energy under mild conditions.^[^ ^5^, ^6^, ^7^, ^8^, ^9^, ^10^, ^11^ ^]^ However, there are still great challenges for simultaneously achieving both high‐efficiency and selectivity of products, owing to the multiple proton‐coupled electron transfer processes affected by complex dynamic factors during CO_2_ reduction, and rational design and development of efficient composite catalysts are urgently desired.^[^ ^12^, ^13^, ^14^, ^15^, ^16^ ^]^

Nanosized Cu catalysts have been leading the catalytic CO_2_ reduction due to their low cost, abundant reserves and specific activity.^[^ ^17^, ^18^, ^19^, ^20^, ^21^, ^22^, ^23^, ^24^ ^]^ Recently, in addition to discrete 0D Cu nanoparticles,^[^ ^25^, ^26^, ^27^, ^28^, ^29^, ^30^, ^31^ ^]^ Cu nanowires (Cu NWs) have been developed for catalyzing the reduction of CO_2_ due to their special surface and interface properties, and excellent electron conductivity as 1D nanometals.^[^ ^32^, ^33^, ^34^, ^35^, ^36^ ^]^ However, the relatively low stability and the uncontrolled competing hydrogen evolution reaction (HER) limit the selectivity of target products and hinder further applications of Cu NWs.^[^ ^37^ ^]^

As a new type of crystalline porous material formed by the assembly of metal ions/metal clusters and organic ligands,^[^ ^38^, ^39^, ^40^ ^]^ metal–organic frameworks (MOFs) provide an ideal platform for precisely modifying surfaces and interfaces, and constructing composite/synergetic catalysts, due to their ultrahigh porosity and surface areas, designable structures, microenvironments of pores/channels at the molecular level, abundant functional groups, and ability to capture reactant molecules with high selectivity.^[^ ^41^, ^42^, ^43^, ^44^, ^45^ ^]^ Herein, we used Cu NWs as the “electron channel” to conduct, transmit and directionally supply electrons for improving the current efficiency, and rationally grew a layer of MOFs on the surface of Cu NWs as the “molecule channel” to auto‐concentrate CO_2_ molecules, control the entrance of H_2_O molecules, and switch the molecular productions for optimizing the stability, conversion rate and the selectivity. Consequently, a brand‐new family of heterowires, Cu NW@MOFs, were constructed, which acted as switchable 1D dual‐channel catalysts with superior stability to accurately manipulate the CO_2_RR production from relatively pure CO to complete syngas with broadly variable CO/H_2_ ratios from 1:1 to 1:3.2. To further illustrate the role of these two channels in the heterowire catalysts, a photocatalytic heterowire, Cu NWs@HKUST‐1, was constructed by using the semiconducting MOF cover as both molecule channel and photoelectric converter, which exhibited the first 1D/1D‐type Mott–Schottky heterojunction that significantly boosted the activity for photocatalytic CO_2_ reduction to CO, confirming the molecule and electron communication between the dual channels of the heterowire electro‐/photocatalysts. This contribution provides a reliable solution and materials for both CO_2_ management and green syngas‐supply toward vast synthetic requirements with all‐round superiorities including stability, efficiency, and product economy.

## 2 Results and Discussion

### 2.1 Construction of Cu NWs@ZIF‐8 Heterowires

**Figure 1:**
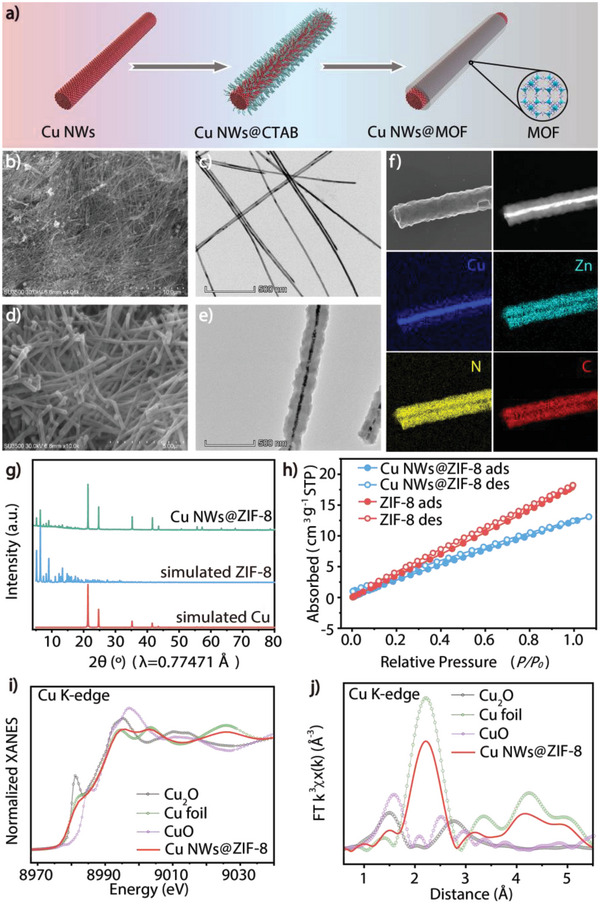
Preparation and characterization of Cu NW@MOF 1D heterowire catalysts. a) Schematic illustration for the synthesis of Cu NWs@MOF composite. b) SEM image of as‐synthesized Cu NWs. c) TEM image of as‐synthesized Cu NWs. d) SEM image of as‐synthesized Cu NWs@ZIF‐8. e) TEM image of as‐synthesized Cu NWs@ZIF‐8. f) HAADF‐STEM image and elemental maps (C, N, Cu, Zn) of Cu NWs@ZIF‐8. g) Synchrotron PXRD (*λ* = 0.77471 Å) of as‐prepared Cu NWs@ZIF‐8. h) CO_2_ uptake amount of ZIF‐8 and Cu NWs@ZIF‐8 at 298 K. i) Normalized Cu K‐edge XANES spectra of Cu NWs@ZIF‐8. j) R‐space Cu K‐edge EXAFS spectra of Cu NWs@ZIF‐8.

Cu NWs were hydrothermally synthesized according to reported approaches.^[^ ^46^, ^47^ ^]^ Then, a layer of ZIF‐8 was in‐situ wrapped on the surface of the Cu NWs with hexadecyltrimethylammonium bromide (CTAB)^[^ ^48^, ^49^, ^50^ ^]^ as surfactants (**Figure** **1**a). For a long time, there has been a synthetic bottleneck hindering the encapsulation of Cu nanoobjects into MOFs to form a well‐defined composite compared with other metal nanocrystals (Pt, Pd, Au, etc.) because nanosized Cu is easily oxidized and dissolved in acidic solutions under conventional synthetic conditions.^[^ ^51^, ^52^, ^53^ ^]^ To coat MOF ZIF‐8 onto Cu NWs, the fresh Cu NWs were first dispersed in degassed methanol, and dual‐role CTAB was added as both a surfactant and protectant with further ultrasonic dispersion. Then, solutions of dimethylimidazole ligand and zinc nitrate in degassed methanol were sequentially added to the above dispersion under N_2_. Finally, a light pink sample of Cu NWs@ZIF‐8 was obtained after 1 h of reaction by centrifugation under the protection of an inert gas and was dried under dynamic vacuum.

Structure and morphology of the as‐prepared Cu NWs and Cu NWs@ZIF‐8 were characterized by powder X‐ray diffraction (PXRD), synchrotron PXRD, scanning electron microscopy (SEM) and transmission electron microscopy (TEM). The diffraction pattern (Figure S1, Supporting Information) of the as‐prepared red‐brown Cu NWs shows three sharp peaks with high diffraction intensities at 43.3°, 50.4°, and 74.1°, corresponding to (111), (200), and (220) of Cu (PDF#04‐0836). The SEM and low‐resolution TEM images confirm the uniform nanowire structure of Cu NWs with a diameter of 22.1±3.5 nm and a length of ≈10 µm (Figure 1b,c; Figures S2 and S3, Supporting Information). The X‐ray photoelectron spectroscopy (XPS) spectrum (Figure S4a,b, Supporting Information) of the as‐prepared Cu NWs shows two peaks at binding energies of 932.6 and 952.5 eV, which could be attributed to Cu2p in Cu/Cu_2_O. In addition, there are peaks at binding energies of 934.4 and 954.4 eV accompanied by satellite peaks at ≈941.9, 944.1, and 962.5 eV, which are assigned to CuO,^[^ ^54^ ^]^ indicating that the surface of the Cu NWs is easily oxidized in air. Nevertheless, this oxidized surface of Cu NWs is easily recovered to Cu under reaction conditions through both XPS of a sample etched at a depth of 2 nm and CV activation within 50 cycles (Figure S4c,d, Supporting Information).

To characterize the fine structure of the Cu NWs@ZIF‐8 composite, synchrotron PXRD (*λ* = 0.77471 Å) was used to analyze the phase structure. Benefiting from the high resolution of synchrotron PXRD (Figure 1g), characteristic diffraction peaks of ZIF‐8 were clearly observed in the range of 4°–20°, the intense Cu NWs peaks were maintained, and no other side peaks were observed, indicating the successful combination of Cu NWs with ZIF‐8 and the well‐preserved crystalline phases of both Cu NWs and the MOF cover. Infrared spectroscopy confirmed that there was no CTAB residue in the Cu NWs@ZIF‐8 composite (Figure S5, Supporting Information). X‐ray absorption fine structure (XAFS) measurements of the heterowire at the Cu K‐edges exhibit characteristics the same as those of Cu foil compared to Cu_2_O and CuO in the XANES spectra (Figure 1i), indicating that the Cu NWs remain metallic in Cu NWs@ZIF‐8. The Fourier transform Cu K‐edge EXAFS curve of Cu NWs@ZIF‐8 (Figure 1j) displays intense peaks located around 2.2 Å corresponding to the Cu–Cu bond, confirming the effective protection of the Cu NWs from oxidation by the MOF cover, which guarantee the perfect long‐term stability of these dual‐channel catalysts.

The morphology of Cu NWs@ZIF‐8 was characterized through a combination of microscopy techniques (SEM and TEM). The SEM images show that the covered Cu NWs are well dispersed and that a layer of ZIF‐8 has been uniformly grown on the Cu NWs to form a skin‐core structure (Figure 1d,e and Figure S6, Supporting Information). A secondary electron imaging (SEI) image demonstrates the smooth surface of a Cu NWs@ZIF‐8 composite composed of polycrystalline ZIF‐8 (Figure 1f and Figure S7, Supporting Information). In the TEM images, the Cu NWs core and the ZIF‐8 shell are clearly illustrated (Figure 1e and Figure S7, Supporting Information). Elemental mapping analysis was carried out by means of TEM in scanning transmission electron microscopy (STEM) mode to reveal the spatial distribution of different elements in Cu NWs@ZIF‐8 (Figure 1f). Extending outward from the center along the radial direction of the nanowire, the Cu elemental mapping represents Cu NWs, and Zn element mapping (including C and N elemental mapping) represents the ZIF‐8 layer. The thickness of the ZIF‐8 shell is ≈40 nm.

### 2.2 Electrocatalytic Reduction of CO_2_ to CO with the Heterowire Cu NWs@ZIF‐8

**Figure 2:**
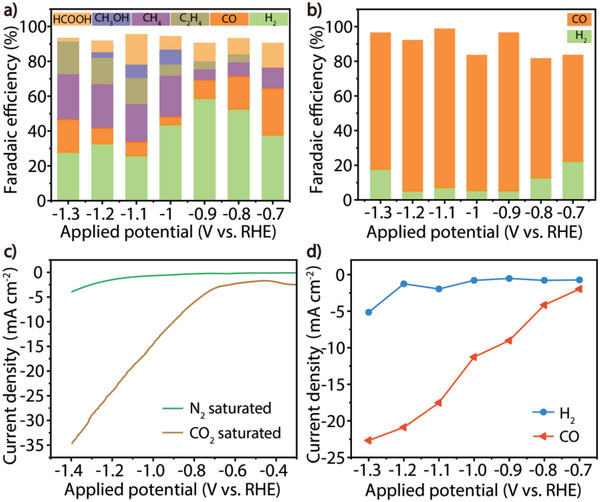
Catalytic activity and stability of the dual‐channel heterowire Cu NWs@ZIF‐8 for electrocatalytic reduction of CO_2_. a) FE of the main products for Cu NWs under different applied reduction potentials. b) FE of the main products for Cu NWs@ZIF‐8 under different applied reduction potentials. c) LSV of Cu NWs@ZIF‐8 in N_2_ and CO_2_ saturated 0.1 m KHCO_3_ electrolyte. d) Partial current densities toward CO and H_2_ for Cu NWs@ZIF‐8 in 0.1 m KHCO_3_.

The electrochemical characterization of the dual‐channel heterowire catalyst Cu NWs@ZIF‐8 and its electrocatalytic CO_2_ reduction performance was carried out in a commercial gas‐tight H‐type flow cell with a standard three‐electrode system at room temperature under atmospheric pressure. A glassy carbon electrode was used as the working electrode, an Ag/AgCl electrode with a saturated KCl filling solution was used as the reference electrode, and a platinum wire electrode was used as the counter electrode. Linear sweep voltammetry (LSV) in a CO_2_‐saturated 0.1 m potassium bicarbonate (KHCO_3_) electrolyte (pH = 6.8) was used to initially determine the electrochemical activity and the onset reduction potential of the catalyst. Current densities were normalized by the geometric surface. Fifty cycles of cyclic voltammetry (CV) sweeps were conducted before LSV was performed. The LSV curves of Cu NWs and Cu NWs@ZIF‐8 (**Figure** **2**c) indicate that the onset potential was more negative than −0.60 V versus reversible hydrogen electrode (RHE). Considering that a considerable current density was required to evaluate the catalytic activity of the catalyst, a reduction potential range of −0.7 to −1.3 V_RHE_ was used in

the electrocatalytic reduction of CO_2_. As shown in Figure 2a, Cu NWs exhibited a wide distribution of reduction products under the applied reduction potential. When the reduction potential was more positive than −0.9 V_RHE_, H_2_ was generated with a faradaic efficiency (FE) over 40%, and a mixture of CO, C_2_H_4_, and CH_4_ in the gas phase and HCOOH in the liquid phase were obtained from the CO_2_RR, with a poor total FE. Although the HER was suppressed under a more negative reduction potential (−1.0 V_RHE_), the minimum FE still reached 20% at −1.1 V_RHE_, and the total FE of the CO_2_RR was ≈95%, with a product distribution of 22.3% C_2_H_4_, 15.0% CH_4_, 7.7% CO and a small amount of CH_3_OH. This product distribution is similar to that obtained for the CO_2_RR on polycrystalline metallic nano‐copper reported previously.^[^ ^55^, ^56^ ^]^ The LSV curve of ZIF‐8 (Figure S8, Supporting Information) shows negligible activity to electrocatalytic CO_2_ reduction.

The same reduction potential range was used to evaluate the activity of the Cu NWs@ZIF‐8 according to the comparable onset reduction potentials of Cu NWs (Figure 2b). As clearly shown in Figure 2b, both the FE and selectivity of CO_2_RR were greatly improved for the composite Cu NWs@ZIF‐8 compared with Cu NWs. The onset reduction potential to CO was significantly reduced, and an FE of CO more than 60% was obtained at −0.7 V_RHE_. CO was the only carbon product in the entire test potential range. The FE of CO was increased to 65.2–91.3%, and the competitive HER was effectively suppressed as low as 5.2%. At −1.1 V_RHE_, ≈100% total FE and 91.3% CO were achieved (Figure 2b). It is worth noting that when the current density was normalized to the unit mass of Cu (Figure 2d; Table S1, Supporting Information), the equivalent CO yield was increased by 65 times along with the perfect selectivity. Subsequently, another important parameter for performance of the electrocatalytic reduction of CO_2_ catalyst, the long‐term stability of the heterowire catalyst was evaluated at −1.1 V_RHE_. The HER was well suppressed during the whole measurement, and Cu NWs@ZIF‐8 exhibited excellent cycle stability for both FE and selectivity of CO as well as the total current density even after 24 hours (Figure S9, Supporting Information), which was superior than all the other Cu‐based CO_2_RR electrocatalysts (2–15 h; Table S2, Supporting Information), indicating the reliable protective effect of the ZIF‐8 cover around the Cu NWs.

SEM images show that the catalyst maintains its original morphological structure after the reaction (Figure S10, Supporting Information). In contrast, Cu NWs is easily inactivated under the same reaction conditions, with the extension of the reaction time, the FE of H_2_ increased rapidly after 7 h (Figure S11, Supporting Information). This may be caused by surface reconstruction of the Cu NWs during the reaction.^[^ ^55^, ^57^ ^]^ The SEM images (Figure S12, Supporting Information) of the Cu NWs after the reaction shows that there are many small particles on the surface compared to the smooth surface of the fresh sample, and the carbon products of CO change much at a low level. This proves that our synthesis strategy is very effective in improving activity and maintaining stability in CO_2_RR.

**Figure 3:**
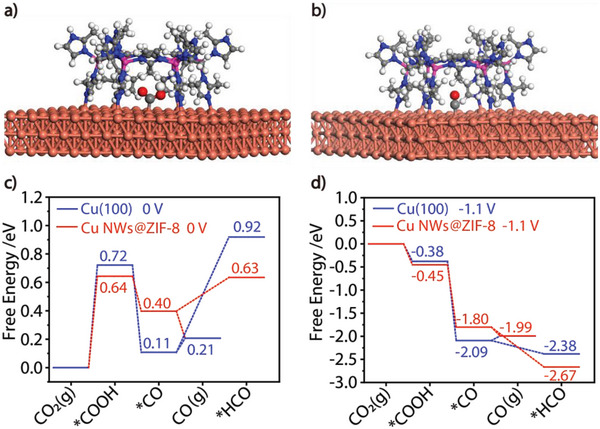
DFT calculations. a) DFT optimized structure of intermediate *COOH adsorbed on Cu NWs@ZIF‐8. b) DFT optimized structure of intermediate *CO adsorbed on Cu NWs@ZIF‐8. The orange, pink, grey, blue and red spheres represent Cu, Zn, C, N, and O atoms, respectively. c) Calculated binding energies (in eV) of key reaction intermediates on Cu(100) and Cu NWs@ZIF‐8 at a potential of 0 V. d) Calculated binding energies (in eV) of key reaction intermediates on Cu(100) and Cu NWs@ZIF‐8 at a potential of −1.1 V_RHE_.

Density functional theory (DFT) calculations were performed to insight into the activity and selectivity of the Cu NWs@MOF dual‐channel catalytic system for CO_2_RR. During the electrocatalytic reduction of CO_2_ by Cu NWs@ZIF‐8, we speculate that the catalytic active center of the 1D composite is located at the interface between Cu NWs and ZIF‐8 since the activity of ZIF‐8 is negligible compared to that of Cu NWs (Figure S8, Supporting Information). Therefore, the binding energy of key reaction intermediates *COOH and *CO on Cu(100) of Cu NWs and on the interface of Cu NWs and ZIF‐8 were calculated, respectively. **Figure** **3**a,b shows the optimized structures used in DFT calculations and the energetically most favorable adsorption configurations of the intermediates *COOH and *CO on Cu NWs@ZIF‐8. As shown in Figure 3c, the formation of key intermediates *COOH and *CO in Cu NWs@ZIF‐8 composite system is the dominant path compared with the Cu(100) surface (Figure S13a–g, Supporting Information), and more importantly, the desorption of *CO to form CO is thermodynamically favorable. Consistent with the experimental measurements, the theoretical calculation results confirm the electron and molecule transmission between Cu NWs and the MOF cover, and clearly illustrate the high activity and excellent CO selectivity of the 1D nanosized Cu NWs@MOF dual‐channel catalyst originating from the cooperation of the electron channel and the molecule channel. Benefiting from the fine conductivity of the Cu NWs electron channel directionally transmitted and continuously supplied electrons to the CO_2_RR process, while the MOF cover acted as the multifunctional molecule channel to protect the Cu NWs, auto‐capture CO_2_ molecules at the interface between Cu NWs and MOF, and maintain a proper CO_2_ concentration surrounding the Cu NWs based on

the good stability and the high BET surface area of ZIF‐8 (Figure S14 and Table S1, Supporting Information), the heterowire Cu NWs@ZIF‐8 catalyst performed as the nanosized dual‐channel composite catalyst to produce relative pure CO with all‐round advantages including high efficiency, good conversion rate, promising selectivity and tremendous long‐term stability.

### 2.3 Dual‐Channel Heterowire Catalysts to Control the CO/H_2_ Ratio of Syngas Products by Co^2+^ Doping

**Figure 4:**
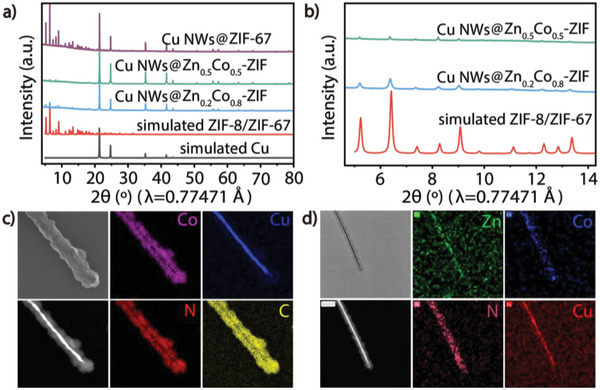
Structural characterization of dual‐channel heterowire Cu NWs@Zn*_x_* Co_1−_ *_x_*‐ZIF and Cu NWs@ZIF‐67. a) Synchrotron PXRD of Cu NWs@ZIF‐67 and Cu NWs@Zn*_x_* Co_1−_ *_x_*‐ZIF (*λ* = 0.77471 Å). b) Local enlargement of (a). c) HAADF‐STEM elemental maps (Cu, Co, C, N) of Cu NWs@ZIF‐67. d) HAADF‐STEM elemental maps (Zn, Co, Cu, N) of Cu NWs@Zn_0.2_Co_0.8_‐ZIF.

Syngas, one of the most important resources as both an energy‐storage fuel and a building block for the synthesis of various industrial products, is a gaseous mixture of CO and H_2_,^[^ ^58^, ^59^ ^]^ Since the electrochemical reduction of CO_2_ is generally performed in aqueous media, HER from the reduction of water or protons is in inevitable rivalry with the CO_2_ conversion. Hence, combining CO_2_ reduction and HER for the production of syngas is believed as the intrinsic nature and one of the most plausible approach for electrocatalytic reduction of CO_2_.^[^ ^5^, ^60^ ^]^ In view of the unique advantages of the ZIF‐8 channel in Cu NWs@ZIF‐8 for reduction of CO_2_ to CO, we attempted to switch the CO/H_2_ ratio to produce syngas from the CO_2_RR by adjusting the composition of the molecule channel. Following the synthesis of Cu NWs@ZIF‐8, Co^2+^ ions together with Zn^2+^ were introduced during the in‐situ coating of the ZIF cover in order to dope Co^2+^ into the molecule channel and to construct a series of Cu NWs@Zn*_x_* Co_1−_ *_x_*‐ZIF type dual‐channel heterowires (Cu NWs@Zn_0.5_Co_0.5_‐ZIF, Cu NWs@Zn_0.4_Co_0.6_‐ZIF, Cu NWs@Zn_0.3_Co_0.7_‐ZIF, and Cu NWs@Zn_0.2_Co_0.8_‐ZIF) by adjusting the Zn^2+^/Co^2+^ ratio in the metal ion precursors. High‐resolution synchrotron PXRD (*λ* = 0.77471 Å) confirmed the setup of Cu NWs@Zn*_x_* Co_1−_ *_x_*‐ZIF heterowires and the integrity of Cu NWs (**Figure** **4**a,b). Electronic microscopy techniques, including SEM, TEM, and HAADF‐STEM elemental mapping further confirmed the successful coating of the hybrid Zn/Co‐ZIF cover with different metallic ratios (Figure 4c,d and Figures S15–S35, Supporting Information), which also revealed the uniform distribution of both Zn and Co elements, indicating the synergy of the hybrid MOF coatings.

**Figure 5:**
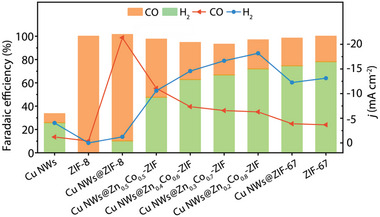
Two‐channel Cu NWs@Zn*_x_* Co_1−_ *_x_*‐ZIF heterowire catalysts electrocatalytic reduction CO_2_ to syngas with different CO/H_2_ ratio at −1.1 V_RHE_.

Considering both energy consumption and current density, the activity of these dual‐channel catalysts were evaluated for the CO_2_RR at a reduction potential of −1.1 V_RHE_. The product distribution, FE values and current densities are summarized in **Figure** **5** and Figure S36 (Supporting Information). As the ratio of Co^2+^ in the MOF channel increased, the main component of the CO_2_RR products changed from CO for Cu NWs@ZIF‐8 to syngas. When the Zn/Co ratio of the catalyst was 0.5:0.5 (Cu NWs@Zn_0.5_Co_0.5_‐ZIF), the CO/H_2_ ratio of the product was ≈1:1 with a promising FE of 97.5%. Further changing the Zn/Co ratio of the hybrid catalysts (Cu NWs@Zn_0.4_Co_0.6_‐ZIF, Cu NWs@Zn_0.3_Co_0.7_‐ZIF, Cu NWs@Zn_0.2_Co_0.8_‐ZIF), the CO/H_2_ ratio in syngas was adjusted from 1:1 to 1:3 with no other by‐product and maintained FE. When the metal ion in the ZIF cover was totally Co^2+^, the heterowire with a ZIF‐67 cover, the Co‐containing analogue of ZIF‐8, resulted a CO/H_2_ ratio of 1:3.2, confirming that Co^2+^ was active to HER and thus acted as an assistant for generating an appropriate amount of H_2_ (Figure S37–S38, Supporting Information) in Cu NWs@Zn*_x_* Co_1−_ *_x_*‐ZIF.^[^ ^61^ ^]^ As a result, a syngas series with different compositions could be selectively produced through CO_2_RR from these switchable heterowire catalysts by either adjusting the MOF components of the molecule channel or controlling the electric parameters of the electron channel, successively satisfying different synthetic requirements for a vast range of essential industrial products.^[^ ^58^, ^59^, ^62^ ^]^

### 2.4 Dual‐Channel Mott–Schottky Heterowire for Photocatalytic Reduction of CO_2_

**Figure 6:**
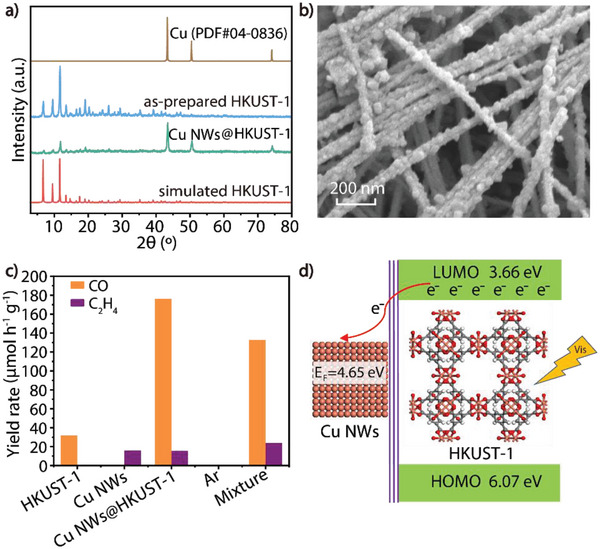
Characterization and tests for photocatalytic CO_2_ reduction of Cu NWs@HKUST‐1. a) XADE of Cu NWs@HKUST‐1. b) SEM of Cu NWs@HKUST‐1. c) Photocatalytic reduction of CO_2_ activity of Cu NWs@HKUST‐1. d) Electronic structures of Cu NWs and the Cu NWs/HUT‐1 based Mott–Schottky contact (*E*_F_: work function).

In order to further extend the newly developed dual‐channel heterowire catalyst family, we raised Cu_3_(BTC)_2_ (BTC = 1,3,5‐benzene tricarboxylate) (HKUST‐1), a semiconducting MOF,^[^ ^63^ ^]^ as the cover of Cu NWs instead of ZIF for introducing the photocatalytic activity besides the molecule and electron channels. XRD and SEM (**Figure** **6**a,b) data clearly confirm the successful setup of Cu NWs@HKUST‐1. The activity of Cu NWs@HKUST‐1 for photocatalytic reduction of CO_2_ (1 bar) was carried out at room temperature under visible‐light irradiation (*λ* > 420 nm) (Figure S39, Supporting Information) with water vapor as the only electron donor. Remarkably, promising photocatalytic activity of Cu NWs@HKUST‐1 on CO_2_ reduction was obtained with the yield of CO up to 176.2 µmol h^−1^ g^−1^ in 5 hours accompanied by a small amount of C_2_H_4_, and more importantly, the selectivity of CO reached 92%. Except for CO and C_2_H_4_, no other carbon product was detected in both gas and liquid phases. As a comparison, when solely Cu NWs or HKUST‐1 was used as catalyst, only a small amount of C_2_H_4_ (15.9 µmol h^−1^ g^−1^) or CO (32 µmol h^−1^ g^−1^) was observed, respectively. In addition, as control experiments, no product was detected when using Ar instead of CO_2_, confirming CO_2_ as the carbon source of CO and C_2_H_4_. If Cu NWs and HKUST‐1 were simply physically mixed as catalysts, a much higher H_2_ production was observed, indicating that the catalytic active site is located at the interface between Cu NWs and HKUST‐1.

To further investigate the molecule and electron exchange between the two channels and the catalytic mechanism of Cu NWs@HKUST‐1, Mott–Schottky measurements were conducted at frequencies of 1000, 1500, and 2000 Hz (Figure S40, Supporting Information). The positive slope of the Mott–Schottky patterns illustrated HKUST‐1 as a typical n‐type semiconductor. The flat band position (*V*_fb_) determined from the intersection of Mott−Schottky plots was ≈−0.84 V vs Ag/AgCl. Therefore, the Fermi level of HKUST‐1 (3.86 eV vs AVS) was given by EF = *V*_fb_ (vs SHE) e‐4.44 eV.^[^ ^64^ ^]^ Since the bottom of LUMO in n‐type semiconductors should be approximately equal to the flat‐band potential, the LUMO of HKUST‐1 was estimated as 3.66 eV vs AVS. The band gap of HKUST‐1 was thus estimated as 2.41 eV from the Tauc plot (Figure S41, Supporting Information), and its HOMO was accordingly calculated as 6.07 eV vs AVS. Based on the energy levels of HKUST‐1 in the composite catalyst, the schematic depicting for the band alignments and charge flow at the metal‐MOF interfaces involved in the system has been illustrated in Figure 6d. First, HKUST‐1 undergoes electron‐hole separation under visible light irradiation. Since the Fermi energy level of HKUST‐1 (3.86 eV) is lower than the work function of Cu NWs (4.65 eV),^[^ ^65^ ^]^ the electrons in the conduction band of HKUST‐1 are delivered to Cu NWs driven by Cu NWs‐MOF Schottky barrier, and the interfacial electron exchange generates an electron‐rich Cu NWs together with an electron depletion region on the HKUST‐1 side. Finally, the electron‐rich Cu NWs results in the highest photoactivity of Cu NWs@HKUST‐1 for reducing CO_2_ to CO. It is worthy to mention that as far as we know this is the first example of an 1D/1D type Mott–Schottky heterojunction.^[^ ^65^ ^]^

## 3 Discussion

For the electrocatalytic CO_2_ reduction in a typical H‐type flow cell, the catalytic process mainly includes the following four steps: 1) the continuously bubbling gas phase CO_2_ dissolves in the electrolyte; 2) the dissolved CO_2_ in the electrolyte diffuses to the surface of the catalyst on the electrode; 3) CO_2_ molecules are adsorbed and activated on the catalyst surface to complete the catalytic reaction; and 4) the reaction products are desorbed and dissociated.^[^ ^9^ ^]^ When the MOF cover of the Cu NWs@MOFs heterowire is insulated such as ZIF‐8, the electron channel Cu NW directionally transmits the electrons and may form “micro current” along the Cu NW due to the fine conductivity of copper, as confirmed by the electrochemical impedance spectroscopy (EIS) test of Cu NWs@ZIF‐8 in comparison with ZIF‐8 (Figure S42, Supporting Information). Electrons flowing through the Cu NWs are continuously supplied to the adsorbed CO_2_ molecules, which may have greater opportunity to be reduced in a “long journey” of ≈10 µm along the Cu NWs, leading to much higher electronic utilization efficiency compared with Cu nanoparticles. From a point of view, the dual‐channel Cu NWs@ZIF readily mimic the electric cable consisting of conduction pith and insulation cover, that well regulates the electronic transmission and suppresses the microcurrent power loss.

As the molecule channel, the MOF cover controls the entrance of the reactant molecules and the departure of the product molecules, and brings efficiency, selectivity and stability for the dual‐channel electrocatalytic system: i) the MOF cover significantly improved the dispersion of the Cu NWs and hindered the ultralong Cu NWs from intertwining. ii) The coating of MOF cover significantly enhanced the BET surface area from only 23 m^2^ g^−1^ of the Cu NWs to as high as 800 m^2^ g^−1^ of Cu NWs@ZIF‐8 (Figure S13 and Table S1, Supporting Information), providing a much higher electrochemical active surface area (ECSA) (Figure S43, Supporting Information), which is conducive to obtain a superior CO_2_RR FE and a tenfold increase in CO_2_ converting rate. iii) As illustrated by the hydrophilia tests (Figure S44, Supporting Information), Cu NWs, Cu NWs@ZIF‐8 and Cu NWs@ZIF‐67 shows contact angle of 129.6°, 28.7°, and 26.5° with water, respectively, indicating the hydrophilic character of the MOF cover, which is benefit for transporting electrolytes containing CO_2_ (aq) and protons toward the interface between the Cu NWs and the MOF for the continuous reduction of CO_2_. iv) Furthermore, when using the semiconducting MOF cover such as HKUST‐1 for CO_2_, besides molecule channel MOF even acts as a “fiber converter” to absorb photons and supply electrons to Cu NWs through the Schottky‐effect‐induced electron flow at the interface between Cu NWs and HKUST‐1, and the electron enrichment of Cu NWs can be further enhanced via photoinitiated charge separation at the Cu NWs‐HKUST‐1 interface to significantly promote the efficient photoreduction of CO_2_ to CO.

## 4 Conclusion

In conclusion, a series of 1D dual‐channel Cu NWs@MOFs catalysts were constructed by rationally coating MOFs on the surface of Cu NWs, in which Cu NWs acted an electron channel facilitated electronic transmission while MOFs acted as molecule and/or photon channels to provide an appropriate transport path for both reactants/intermediates and photoelectric conversion. Excellent Faraday efficiency, drastically suppressed HER, and the highest stability of the Cu‐based CO_2_RR electrocatalysts, were obtained by the heterowire catalyst for the electrocatalytic conversion of CO_2_. Moreover, the distribution of the reduction product could be precisely regulated from CO to syngas with different ratio of CO/H_2_ by adjusting the composition of the MOF, which gave the first heterometallic MOF covered 1D composite, Cu NWs@Zn*_x_* Co_1−_ *_x_*‐ZIF. When further switching the MOF cover from insulator to a semiconductor, the first 1D/1D type Mott–Schottky heterojunction, Cu NWs@HKUST‐1, was constructed. Attributed to the Schottky‐effect induced electron flow at the 1D/1D metal–semiconductor interface, Cu NWs@HKUST‐1 exhibits superior catalytic activity in the photocatalytic reduction CO_2_ under visible light irradiation without additional sacrificial agent other than water. The above findings provide a reliable and universal solution for the design of CO_2_ electro‐/photoreduction catalysts. Considering the diversity of MOFs, it is foreseeable that our strategy will play an increasingly important role in both CO_2_ management and energy conversion in the future.

## 5 Experimental Section

### Chemicals and Materials

Copper(II) chloride dihydrate (CuCl_2_·2H_2_O, 99%), Cobalt(II) nitrate hexahydrate (Co(NO_3_)_2_·6H_2_O), d‐(+)‐Glucose (>99.5%), 1‐hexadecylamine (C_16_H_35_N, HDA, 90%), Hexadecyl trimethyl ammonium Bromide (C_19_H_42_BrN, CTAB, 99%), 2‐methylimidazole (C_4_H_6_N_2_, 98%), potassium hydrogen carbonate (KHCO_3_, 99.7%), 1,3,5‐benzenetricarboxylic acid (C_9_H_6_O_6_, H_3_BTC, 99.9%) and polyvinylpyrrolidone (PVP, K13‐18) were all purchased from Sigma‐Aldrich. Zinc nitrate hexahydrate (Zn(NO_3_)_2_·6H_2_O) and cobaltous nitrate hexahydrate (Co(NO_3_)_2_·6H_2_O) was purchased from Acros Organics. KOH and hexane (99.9%) were purchased from Fisher Chemical. All the chemicals were used without purification. The deionized water (18.2 MΩ cm) was used to make the solutions.

### Preparation of Cu NWs

Cu NWs were prepared by an improved hydrothermal method according to the literature. In a typical procedure, 40 mL of an aqueous solution containing 85 mg of CuCl_2_∙2H_2_O, 500 mg of HDA, and 111 mg of d‐(+)‐glucose was vigorously stirred overnight under 45 °C to obtain an emulsion mixture with light‐blue color, followed by sonication for 20 mins. The solution was transferred into a Teflon‐lined stainless‐steel autoclave (100 mL). After 12 h of hydrothermal reaction at 120 °C, the solution was cooled to room temperature and a brown mixture was obtained. The solid product obtained by centrifugation (10 000 rpm, 5 min) and washed three times with water, ethanol, and hexane respectively. Finally, the Cu NWs were stored in 8 mL absolute ethanol.

### Preparation of ZIF‐8

ZIF‐8 was prepared according to the literature.^[^ ^66^, ^67^ ^]^ Briefly, dissolve 372 mg Zn(NO_3_)_2_·6H_2_O in 50 mL methanol to obtain solution A, and dissolve 164 mg 2‐methylimidazole in 50 mL methanol to obtain solution B. Then solution A and solution B were mixed and allowed to react at room temperature for 24 h without stirring. The solid product was collected by centrifugation, washed several times with methanol, and vacuum‐dried overnight at 70 °C.

### Preparation of ZIF‐67

ZIF‐67 was prepared according to literature reported method. In brief, dissolve 364 mg Co(NO_3_)_2_·6H_2_O in 50 mL methanol to obtain solution C, and dissolve 164 mg 2‐methylimidazole in 50 mL methanol to obtain solution D. Then solution C and D were mixed and allowed to react at room temperature for 24 h without stirring. The solid product was collected by centrifugation, washed several times with methanol, and vacuum‐dried overnight at 70 °C.

### Preparation of Cu NWs@ZIF‐8

In a typical synthesis, 2 mL freshly synthesized Cu NWs ethanol dispersion were dispersed in 18 mL degassed methanol solution containing 200 mg CTAB and the solution was degassed for another 10 mins after 20 min of ultrasound. Then 54 mg 2‐methylimidazole and 50 mg Zn(NO_3_)_2_·6H_2_O were dissolved in 5 mL degassed methanol respectively. Finally, methanolic solutions of 2‐methylimidazole and Zn(NO_3_)_2_·6H_2_O were added sequentially into the Cu NWs dispersion under the protection of N_2_ atmosphere. The vial was sealed with a septum and left 1 h at room temperature without stirring. The suspension was collected by centrifugation under air free condition and washed three times with degassed methanol. Solid product obtained by centrifugation and dried under dynamic vacuum overnight at room temperature.

### Preparation of Cu NWs@ZIF‐67

The synthesis method of Cu NWs@ZIF‐67 is similar to that of Cu NWs@ZIF‐8. 2 mL freshly synthesized Cu Nanowires ethanol dispersion were dispersed in 18 mL degassed methanol solution containing 200 mg CTAB and the solution was degassed for another 10 mins after 20 mins of ultrasound. Then 54 mg 2‐methylimidazole and 49 mg Co(NO_3_)_2_·6H_2_O were dissolved in 5 mL degassed methanol respectively. Finally, methanolic solutions of 2‐methylimidazole and Zn(NO_3_)_2_·6H_2_O were added sequentially into the Cu NWs dispersion under the protection of N_2_ atmosphere. The vial was sealed with a septum and left one hour at room temperature without stirring. The suspension was collected by centrifugation under air free condition and washed three times with degassed methanol. Solid product obtained by centrifugation was dried under dynamic vacuum overnight at room temperature.

### Preparation of Cu NWs@Zn*_x_* Co_1−_ *_x_*‐ZIF

The synthesis method of Cu NWs@Zn*_x_* Co_1−_ *_x_*‐ZIF is similar to that of Cu NWs@ZIF‐8. 4 mL freshly synthesized Cu NWs ethanol dispersion were dispersed in 36 mL degassed methanol solution containing 400 mg CTAB and the solution was degassed for another 10 min after 20 min of ultrasound. Then 108 mg 2‐methylimidazole, 50 mg Zn(NO_3_)_2_·6H_2_O and 50 mg Co(NO_3_)_2_·6H_2_O were dissolved in 10 mL degassed methanol respectively. Finally, methanolic solutions of 2‐methylimidazole and Zn(NO_3_)_2_·6H_2_O&Co(NO_3_)_2_·6H_2_O were added sequentially into the Cu NWs dispersion under the protection of N_2_ atmosphere. The vial was sealed with a septum and left 1 h at room temperature without stirring. The suspension was collected by centrifugation under air free condition and washed three times with degassed methanol. Solid product obtained by centrifugation was dried under dynamic vacuum overnight at room temperature. Four samples with different ratio of Zn(NO_3_)_2_·6H_2_O/Co(NO_3_)_2_·6H_2_O (0.5:0.5, 0.4:0.6, 0.3:0.7, 0.2:0.8) were synthesized, and named as Cu NWs@Zn_0.5_Co_0.5_‐ZIF, Cu NWs@Zn_0.4_Co_0.6_‐ZIF, Cu NWs@Zn_0.3_Co_0.7_‐ZIF, Cu NWs@Zn_0.2_Co_0.8_‐ZIF.

### Preparation of HKUST‐1

HKUST‐1 was prepared according to the literature.^[^ ^68^ ^]^ Briefly, dissolve 372 mg Zn(NO_3_)_2_·6H_2_O in 50 mL methanol to obtain solution A, and dissolve 164 mg 2‐methylimidazole in 50 mL methanol to obtain solution B. Then solution A and solution B were mixed and allowed to react at room temperature for 24 h without stirring. The solid product was collected by centrifugation, washed several times with methanol, and vacuum‐dried overnight at 70 °C.

### Preparation of Cu NWs@HKUST‐1

20 mg freshly synthesized Cu NWs were dispersed in 20 mL ethanol containing 5 mg PVP. Then 40 mL ethanol containing 42 mg H_3_BTC was added into the Cu NWs dispersion. Then the mixture was sealed and placed in a 60 °C oven for 6 h. The suspension was collected by centrifugation and washed three times with anhydrous ethanol after reaction. Finally, solid product obtained by centrifugation and dried under dynamic vacuum overnight at 60 °C.

### Characterization of Materials

Morphologies of samples were characterized by scanning electron microscope (SEM, SU3500, HITACHI and MERLIN Compact, ZEISS) and transmission electron microscopy (TEM, JEM‐2800, JEOL and FEI Talos F200X G2, FEI). The crystallographic information was analyzed by X‐ray diffraction (XRD, Ultima IV, Rigaku) equipped with a Cu K*α* radiation source. Specific surface areas, pore volume, and pore size of samples were determined using N_2_ physisorption isotherms at 77 K (ASAP 2020 PLUS Analyzer, Micromeritic). CO_2_ uptake amount were determined at 298 K (ASAP 2020 V4.03, Micromeritic). Metal loadings in the catalysts were determined by inductively coupled plasma optical emission spectrometry (ICP‐OES, SpectroBlue, Spectro). X‐ray photoelectron spectroscopy (XPS, Thermo ESCALAB 250XI, ThermoFisher), The charging effect was corrected using the C1s level (284.8 eV) as the reference. XAFS spectra at the copper K edge and were collected at Australian Synchrotron, ANSTO. Synchrotron powder X‐ray diffraction (Synchrotron PXRD) measurements were measured at PD beamline, Australian Synchrotron, ANSTO with a wavelength of *λ* = 0.77471 Å. UV–vis absorption diffuse reflectance spectra (UV–vis DRS) were recorded on a Cary 100 UV–vis spectrophotometer using an integrating sphere accessory.

### Preparation of Working Electrode

4 mg of freshly prepared dried Cu NWs were dispersed in 1 mL absolute ethanol and ultrasonication for 1 h. Subsequently, 30 µL of Nafion (5 wt%) was added and ultrasonication for another 10 min. 20 µL catalyst ink was dropped onto the surface of a precleaned glassy‐carbon electrode (diameter, 3 mm; area, 0.07 cm^2^) using pipettor and dried under ambient air. For catalysts ZIF‐8, ZIF‐67, Cu NWs@ZIF‐8, and Cu NWs@ZIF‐67, the same method was used to prepare electrodes. The Cu content of catalysts Cu NWs@ZIF‐8 and Cu NWs@ZIF‐67 is 22.2 and 20.7 wt% measured by ICP‐OES, respectively.

### Electrochemical Measurements

All electrochemical measurements were carried out on an Autolab PGSTAT302N potentiostat (Metrohm Autolab) at room temperature. Electrochemical measurements and electrochemical reduction of CO_2_ experiments were conducted by using a customized gastight two‐chamber H‐type cell with a standard three‐electrode system. Glassy carbon electrode coated with catalyst was used as working electrode, Ag/AgCl electrode with a saturated KCl filling solution regularly calibrated by a reversible hydrogen electrode (RHE) was used as the reference electrode and a Pt coil from was used as a counter electrode. Working and reference electrodes were fixed in one chamber and the counter electrode was fixed in the other chamber. Two chambers (Cathode and anode) were separated by a Nafion ion exchange membrane (Nafion 117 membrane, Alfa) and the electrolyte in both chambers are 50 mL 0.1 m KHCO_3_. For cyclic voltammetry (CV) and linear sweep voltammetry (LSV) test, the electrolyte was bubbled with CO_2_ or N_2_ for at least 60 min to get the electrolyte saturated, electrochemical impedance spectroscopy (EIS) was conducted at 0 V vs open‐circuit potential from 0.01 Hz to 100 kHz with an amplitude of 5 mV. The LSV was performed at a scan rate of 10 mV s^−1^ to qualitatively evaluate the catalytic activity and define the constant voltage applied to study the product distribution at each potential.

### The Electrocatalytic CO_2_ Reduction

CO_2_RR performance of catalysts were evaluated in a H‐type flow cell. Before CO_2_RR, the electrolyte was bubbled CO_2_ (Airgas, 99.99%) for 60 min to reach saturation. During the measurement, CO_2_ was continuously purged through a mass flow controller (HORIBA, Japan) into cathode compartment with a flow rate of 10 sccm with stirring at 1000 rpm. The FE was measured by using chronoamperometry for 2–3 h at each applied potential. Gas‐phase products of CO_2_ reduction were quantified by a gas chromatography (GC, Agilent 8860) equipped with equipped with thermal conductivity detectors (TCD) and flame ionization detector (FID). A series of standard gas mixtures (H_2_, CO, CH_4_, C_2_H_4_, and C_3_H_6_, Airgas) were used to establish the calibration curves. 1H‐NMR (Bruker, 400 MHz) was used to detect the liquid phase product. 650 µL of the electrolyte after electrolysis was mixed with 300 µL of D_2_O and 50 µL dimethyl sulfoxide (DMSO) was added as an internal standard. All quantitative data is the average value of three measurements under the same test conditions.

### Photoelectrochemical Measurements

The Mott–Schottky plots (MS) was measured with Autolab PGSTAT302N potentiostat (Metrohm Autolab) by a traditional three‐electrode system. Specifically, a platinum plate was used as the counter electrode and an Ag/AgCl electrode was used as the reference electrode. The preparation of the working electrode was as follows: 5 mg prepared sample was dispersed in the solution of Nafion/ethanol (10 µL/1 mL) and ultrasonicated for 1 h. Then, 80 µL of the mixture was dropped onto an indium tin oxide (ITO) glass substrate with an area of 1.0 cm^2^. The obtained electrode was dried at 120 °C under a vacuum overnight. The electrolyte was 0.5 m sodium sulfate aqueous solution. A 300 W Xe lamp with a 420 nm cutoff filter was used as the light source.

### The Photocatalytic CO_2_ Reduction

The photocatalytic CO_2_ reduction was performed in a 125 mL homemade photoreactor, which was irradiated with a 300 W Xe lamp (HSXF300, Beijing NBET Technology Co., Ltd.) equipped with a 420 nm cutoff filter at 25 °C. Typically, 5 mg catalyst samples were spread on a quartz filter membrane which was placed on the sample holder built in the center of the reacting vessel, then 5 mL pure water was added below the holder, avoiding direct contact with the samples. Then the reactor was filled with 0.1 MPa of high‐purity CO_2_. The gas products after the reaction were quantitatively analyzed by Agilent 8860 gas chromatography equipped with thermal conductivity detectors TCD and FID detector. Helium was used as the carrier gas to detect H_2_, and nitrogen was the carrier gas used to detect other gas products. The liquid phase product was detected by 1H‐NMR (Bruker, 400 MHz).

### DFT Calculations

All the DFT calculations in this work were carried out with a periodic slab model using the Vienna ab initio simulation program.^[^ ^69^, ^70^, ^71^, ^72^ ^]^ The generalized gradient approximation was used with the Perdew–Burke–Ernzerhof^[^ ^73^ ^]^ exchange‐correlation functional. The projector‐augmented wave method^[^ ^74^, ^75^ ^]^ was utilized to describe the electron‐ion interactions and the cut‐off energy for the plane‐wave basis set was 450 eV. To illustrate the long‐range dispersion interactions between the adsorbates and catalysts. Brillouin zone integration was accomplished using a 3×3×1 Monkhorst–Pack k‐point mesh. All the adsorption geometries were optimized using a force‐based conjugate gradient algorithm. For the modelling of Cu(100), the crystal structure was optimized; Cu(100) were modelled with a periodic three‐layer p(3×3) model with the two lower layers fixed and the two upper layers relaxed. Cu NWs@ZIF‐8 was modelled with a periodic three‐layer p(10×10) model. In order to reduce the amount of calculation, a window of the ZIF‐8 cage was chosen to grow on the Cu(100) surface for modeling. The atomic coordinates of the optimized models are provided in Figure S12 (Supporting Information).

## Conflict of Interest

The authors declare no conflict of interest.

## Supporting information

Supporting Information

## Data Availability

The data that support the findings of this study are available in the supplementary material of this article.

## References

[1] S. Chu , A. Majumdar , Nature 2012, 488, 294. 22895334 10.1038/nature11475

[2] M. Aresta , A. Dibenedetto , A. Angelini , Chem. Rev. 2014, 114, 1709. 24313306 10.1021/cr4002758

[3] S. M. Jordaan , C. Wang , Nat. Catal. 2021, 4, 915.

[4] Q. Liu , L. Wu , R. Jackstell , M. Beller , Nat. Commun. 2015, 6, 5933. 25600683 10.1038/ncomms6933

[5] W. Zhou , K. Cheng , J. Kang , C. Zhou , V. Subramanian , Q. Zhang , Y. Wang , Chem. Soc. Rev. 2019, 48, 3193. 31106785 10.1039/c8cs00502h

[6] Y. Y. Birdja , E. Pérez‐Gallent , M. C. Figueiredo , A. J. Göttle , F. Calle‐Vallejo , M. T. M. Koper , Nat. Energy 2019, 4, 732.

[7] C. Chen , J. F. K. Kotyk , S. W. Sheehan , Chem 2018, 11, 2571.

[8] Q. Zhu , C. J. Murphy , L. R. Baker , J. Am. Chem. Soc. 2022, 144, 2829. 35137579 10.1021/jacs.1c11500

[9] A. A. Tountas , G. A. Ozin , M. M. Sain , Nat. Catal. 2021, 4, 934.

[10] S. Xu , E. A. Carter , Chem. Rev. 2019, 119, 6631. 30561988 10.1021/acs.chemrev.8b00481

[11] A. Wagner , C. D. Sahm , E. Reisner , Nat. Catal. 2020, 3, 775.

[12] M. B. Ross , P. D. Luna , Y. Li , C.‐T. Dinh , D. Kim , P. Yang , E. H. Sargent , Nat. Catal. 2019, 2, 648.

[13] Z. W. Seh , J. Kibsgaard , C. F. Dickens , I. Chorkendorff , J. K. Nørskov , T. F. Jaramillo , Science 2017, 355, 146. 10.1126/science.aad4998 28082532

[14] Y. Hori , H. Wakebe , T. Tsukamoto , O. Koga , Electrochim. Acta 1994, 39, 1833.

[15] J. Qiao , Y. Liu , F. Hong , J. Zhang , Chem. Soc. Rev. 2014, 43, 631. 24186433 10.1039/c3cs60323g

[16] E. L. Clark , C. Hahn , T. F. Jaramillo , A. T. Bell , J. Am. Chem. Soc. 2017, 139, 15848. 28988474 10.1021/jacs.7b08607

[17] M. B. Gawande , A. Goswami , F.‐X. Felpin , T. Asefa , X. Huang , R. Silva , X. Zou , R. Zboril , R. S. Varma , Chem. Rev. 2016, 116, 3722. 26935812 10.1021/acs.chemrev.5b00482

[18] S. Nitopi , E. Bertheussen , S. B. Scott , X. Liu , A. K. Engstfeld , S. Horch , B. Seger , I. E. L. Stephens , K. Chan , C. Hahn , J. K. Nørskov , T. F. Jaramillo , I. Chorkendorff , Chem. Rev. 2019, 119, 7610. 31117420 10.1021/acs.chemrev.8b00705

[19] W. Wang , L. Wang , W. Su , Y. Xing , J. CO2 Util. 2022, 61, 102056.

[20] G. Wang , Y. Wu , Z. Li , Z. Lou , Q. Chen , Y. Li , D. Wang , J. Mao , Angew. Chem., Int. Ed. 2023, 62, e202218460. 10.1002/anie.202218460 36749548

[21] D.‐L. Meng , M.‐D. Zhang , D.‐H. Si , M.‐J. Mao , Y. Hou , Y.‐B. Huang , R. Cao , Angew. Chem., Int. Ed. 2021, 60, 25485. 10.1002/anie.202111136 34533874

[22] R. Xu , D.‐H. Si , S.‐S. Zhao , Q.‐J. Wu , X.‐S. Wang , T.‐F. Liu , H. Zhao , R. Cao , Y.‐B. Huang , J. Am. Chem. Soc. 2023, 145, 8261. 36976930 10.1021/jacs.3c02370

[23] Q.‐J. Wu , D.‐H. Si , Q. Wu , Y.‐L. Dong , R. Cao , Y.‐B. Huang , Angew. Chem., Int. Ed. 2023, 62, e202215687. 10.1002/anie.202215687 36424351

[24] Y. Wang , X. Zheng , D. Wang , Nano Res. 2022, 15, 1730.

[25] D. Kim , C. S. Kley , Y. Li , P. Yang , Proc. Natl. Acad. Sci. USA 2017, 114, 10560. 28923930 10.1073/pnas.1711493114 PMC5635920

[26] A. Loiudice , P. Lobaccaro , E. A. Kamali , T. Thao , B. H. Huang , J. W. Ager , R. Buonsanti , Angew. Chem., Int. Ed. 2016, 55, 5789. 10.1002/anie.201601582 27059162

[27] A. Bagger , W. Ju , A. S. Varela , P. Strasser , J. Rossmeisl , ACS Catal. 2019, 9, 7894.

[28] J. Huang , M. Mensi , E. Oveisi , V. Mantella , R. Buonsanti , J. Am. Chem. Soc. 2019, 141, 2490. 30657662 10.1021/jacs.8b12381

[29] X. Chang , T. Wang , Z. Zhao , P. Yang , J. Greeley , R. Mu , G. Zhang , Z. Gong , Z. Luo , J. Chen , Y. Cui , G. Ozin , J. Gong , Angew. Chem., Int. Ed. 2018, 57, 15415. 10.1002/anie.201805256 30329205

[30] J.‐J. Velasco‐Vélez , T. Jones , D. Gao , E. Carbonio , R. Arrigo , C.‐J. Hsu , Y.‐C. Huang , C.‐L. Dong , J.‐M. Chen , J.‐F. Lee , P. Strasser , B. R. Cuenya , R. Schlögl , A. Knop‐Gericke , C.‐H. Chuang , ACS Sustainable Chem. Eng. 2019, 7, 1485.

[31] T.‐C. Chou , C.‐C. Chang , H.‐L. Yu , W.‐Y. Yu , C.‐L. Dong , J.‐J. Velasco‐Vélez , C.‐H. Chuang , L.‐C. Chen , J.‐F. Lee , J.‐M. Chen , H.‐L. Wu , J. Am. Chem. Soc. 2020, 142, 2857. 31955572 10.1021/jacs.9b11126

[32] C. W. Li , M. W. Kanan , J. Am. Chem. Soc. 2012, 134, 7231. 22506621 10.1021/ja3010978

[33] D. Wakerley , S. Lamaison , F. Ozanam , N. Menguy , D. Mercier , P. Marcus , M. Fontecave , V. Mougel , Nat. Mater. 2019, 18, 1222. 31384032 10.1038/s41563-019-0445-x

[34] D. Zhang , R. Wang , M. Wen , D. Weng , X. Cui , J. Sun , H. Li , Y. Lu , J. Am. Chem. Soc. 2012, 134, 14283. 22813082 10.1021/ja3050184

[35] H. Zhang , Y. Zhang , Y. Li , S. Ahn , G. T. R. Palmore , J. Fu , A. A. Peterson , S. Sun , Nanoscale 2019, 11, 12075. 31215587 10.1039/c9nr03170g

[36] C. Choi , S. Kwon , T. Cheng , M. Xu , P. Tieu , C. Lee , J. Cai , H. M. Lee , X. Pan , X. Duan , W. A. Goddard III , Y. Huang , Nat. Catal. 2020, 3, 804.

[37] M. Ma , K. Djanashvili , W. A. Smith , Angew. Chem., Int. Ed. 2016, 55, 6680. 10.1002/anie.201601282 27098996

[38] H. Furukawa , K. E. Cordova , M. O'Keeffe , O. M. Yaghi , Science 2013, 341, 1230444. 23990564 10.1126/science.1230444

[39] H.‐C. Zhou , S. Kitagawa , Chem. Soc. Rev. 2014, 43, 5415. 25011480 10.1039/c4cs90059f

[40] G. Maurin , C. Serre , A. Cooper , G. Férey , Chem. Soc. Rev. 2017, 46, 3104. 28561090 10.1039/c7cs90049j

[41] R.‐Z. Zhang , B.‐Y. Wu , Q. Li , L.‐L. Lu , W. Shi , P. Cheng , Coordin. Chem. Rev. 2020, 422, 213436.

[42] J. Li , H. Huang , W. Xue , K. Sun , X. Song , C. Wu , L. Nie , Y. Li , C. Liu , Y. Pan , H.‐L. Jiang , D. Mei , C. Zhong , Nat. Catal. 2021, 4, 719.

[43] Q.‐J. Wu , J. Liang , Y.‐B. Huang , R. Cao , Acc. Chem. Res. 2022, 55, 2978. 36153952 10.1021/acs.accounts.2c00326

[44] C. He , J. Liang , Y.‐H. Zou , J.‐D. Yi , Y.‐B. Huang , R. Cao , Natl. Sci. Rev. 2022, 9, nwab157. 35822067 10.1093/nsr/nwab157 PMC9270066

[45] J.‐D. Yi , R. Xie , Z.‐L. Xie , G.‐L. Chai , T.‐F. Liu , R.‐P. Chen , Y.‐B. Huang , R. Cao , Angew. Chem., Int. Ed. 2020, 59, 23641. 10.1002/anie.202010601 32926542

[46] D. V. R. Kumar , I. Kim , Z. Zhong , K. Kim , D. Lee , J. Moon , Phys. Chem. Chem. Phys. 2014, 16, 22107. 25209426 10.1039/c4cp03880k

[47] M. Jin , G. He , H. Zhang , J. Zeng , Z. Xie , Y. Xia , Angew. Chem., Int. Ed. 2011, 50, 10560. 10.1002/anie.201105539 21928444

[48] P. Hu , J. Zhuang , L.‐Y. Chou , H. K. Lee , X. Y. Ling , Y.‐C. Chuang , C.‐K. Tsung , J. Am. Chem. Soc. 2014, 136, 10561. 25007206 10.1021/ja5048522

[49] M.‐S. Yao , W.‐X. Tang , G.‐E. Wang , B. Nath , G. Xu , Adv. Mater. 2016, 28, 5229. 27153113 10.1002/adma.201506457

[50] B. Li , J.‐G. Ma , P. Cheng , Angew. Chem., Int. Ed. 2018, 57, 6834. 10.1002/anie.201801588 29520923

[51] L.‐I. Hung , C.‐K. Tsung , W. Huang , P. Yang , Adv. Mater. 2010, 22, 1910. 20526993 10.1002/adma.200903947

[52] B. Rungtaweevoranit , J. Baek , J. R. Araujo , B. S. Archanjo , K. M. Choi , O. M. Yaghi , G. A. Somorjai , Nano Lett. 2016, 16, 7645. 27960445 10.1021/acs.nanolett.6b03637

[53] I. Luz , A. Loiudice , D. T. Sun , W. L. Queen , R. Buonsanti , Chem. Mater. 2016, 28, 3839.

[54] Z. Lyu , M. Xie , E. Aldama , M. Zhao , J. Qiu , S. Zhou , Y. Xia , ACS Appl. Nano Mater. 2019, 2, 1533.

[55] R. Reske , H. Mistry , F. Behafarid , B. R. Cuenya , P. Strasser , J. Am. Chem. Soc. 2014, 136, 6978. 24746172 10.1021/ja500328k

[56] G. L. D. Gregorio , T. Burdyny , A. Loiudice , P. Iyengar , W. A. Smith , R. Buonsanti , ACS Catal. 2020, 10, 4854. 32391186 10.1021/acscatal.0c00297 PMC7199425

[57] S. H. Lee , J. C. Lin , M. Farmand , A. T. Landers , J. T. Feaster , J. E. A. Acosta , J. W. Beeman , Y. Ye , J. Yano , A. Mehta , R. C. Davis , T. F. Jaramillo , C. Hahn , W. S. Drisdell , J. Am. Chem. Soc. 2021, 143, 588. 33382947 10.1021/jacs.0c10017

[58] S. R. Foit , I. C. Vinke , L. G. J. deHaart , R. A. Eichel , Angew. Chem., Int. Ed. 2017, 56, 5402. 10.1002/anie.201607552 27714905

[59] F. Jiao , J. Li , X. Pan , J. Xiao , H. Li , H. Ma , M. Wei , Y. Pan , Z. Zhou , M. Li , S. Miao , J. Li , Y. Zhu , D. Xiao , T. He , J. Yang , F. Qi , Q. Fu , X. Bao , Science 2016, 351, 1065. 26941314 10.1126/science.aaf1835

[60] S. Hernández , M. A. Farkhondehfal , F. Sastre , M. Makkee , G. Saracco , N. Russo , Green Chem. 2017, 19, 2326.

[61] D. Saliba , M. Ammar , M. Rammal , M. Al‐Ghoul , M. Hmadeh , J. Am. Chem. Soc. 2018, 140, 1812. 29302958 10.1021/jacs.7b11589

[62] Y. Chen , J. Wei , M. S. Duyar , V. V. Ordomsky , A. Y. Khodakov , J. Liu , Chem. Soc. Rev. 2021,50, 2337. 33393529 10.1039/d0cs00905a

[63] J. Liu , Y. Wang , A. I. Benin , P. Jakubczak , R. R. Willis , M. D. LeVan , Langmuir 2010, 26, 14301. 20707342 10.1021/la102359q

[64] L. Zhang , J. Ran , S.‐Z. Qiao , M. Jaroniec , Chem. Soc. Rev. 2019, 48, 5184. 31432886 10.1039/c9cs00172g

[65] D. Xu , S.‐N. Zhang , J.‐S. Chen , X.‐H. Li , Chem. Rev. 2023,123, 1. 36342422 10.1021/acs.chemrev.2c00426

[66] X.‐C. Huang , Y.‐Y. Lin , J.‐P. Zhang , X.‐M. Chen , Angew. Chem. 2006, 118, 1587;

[67] K. S. Park , Z. Ni , A. P. Côté , J. Y. Choi , R. Huang , F. J. Uribe‐Romo , H. K. Chae , M. O'Keeffe , O. M. Yaghi , Proc. Natl. Acad. Sci. USA 2006, 103, 10186. 16798880 10.1073/pnas.0602439103 PMC1502432

[68] S. S.‐Y. Chui , S. M.‐F. Lo , J. P. H. Charmant , A. Guy Orpen , I. D. Williams , Science 1999, 283, 2050. 10024237 10.1126/science.283.5405.1148

[69] G. Kresse , J. Furthmüller , Phys. Rev. B 1996, 54, 11169. 10.1103/physrevb.54.11169 9984901

[70] G. Kresse , J. Furthmüller , Comput. Mater. Sci. 1996, 6, 15.

[71] G. Kresse , J. Hafner , Phys. Rev. B 1993, 47, 558. 10.1103/physrevb.47.558 10004490

[72] G. Kresse , J. Hafner , Phys. Rev. B 1994, 49, 14251. 10.1103/physrevb.49.14251 10010505

[73] J. P. Perdew , K. Burke , M. Ernzerhof , Phys. Rev. Lett. 1996, 77, 3865. 10062328 10.1103/PhysRevLett.77.3865

[74] G. Kresse , D. Joubert , Phys. Rev. B 1999, 59, 1758.

[75] P. E. Blöchl , Phys. Rev. B 1994, 50, 17953. 10.1103/physrevb.50.17953 9976227

