# Supplementary material for: Ultrastable Cu‐Based Dual‐Channel Heterowire for the Switchable Electro‐/Photocatalytic Reduction of CO2
Source: Adv Sci (Weinh). 2023 Jul 2;10(26):2302881. doi: 10.1002/advs.202302881 (PMC10502641; doi:10.1002/advs.202302881)
Supplement: Supplementary file 1 — Supporting Information [file ADVS-10-2302881-s001.pdf]

ADVANCED SCIENCE
Open Access

Supporting Information

for *Adv. Sci.*, DOI 10.1002/advs.202302881

Ultrastable Cu-Based Dual-Channel Heterowire for the Switchable Electro-/Photocatalytic Reduction of CO_2

Bo Li, Xiao Liu, Bin Lei, Haiqiang Luo, Xize Liu, Hengzhi Liu, Qinfen Gu, Jian-Gong Ma and Peng Cheng**

Supporting Information

Ultra-stable Cu-based dual-channel heterowire for the switchable electro-/photo-catalytic reduction of CO_2

Bo Li, Xiao Liu, Bin Lei, Haiqiang Luo, Xize Liu, Hengzhi Liu, Qinfen Gu, Jian-Gong Ma and Peng Cheng**

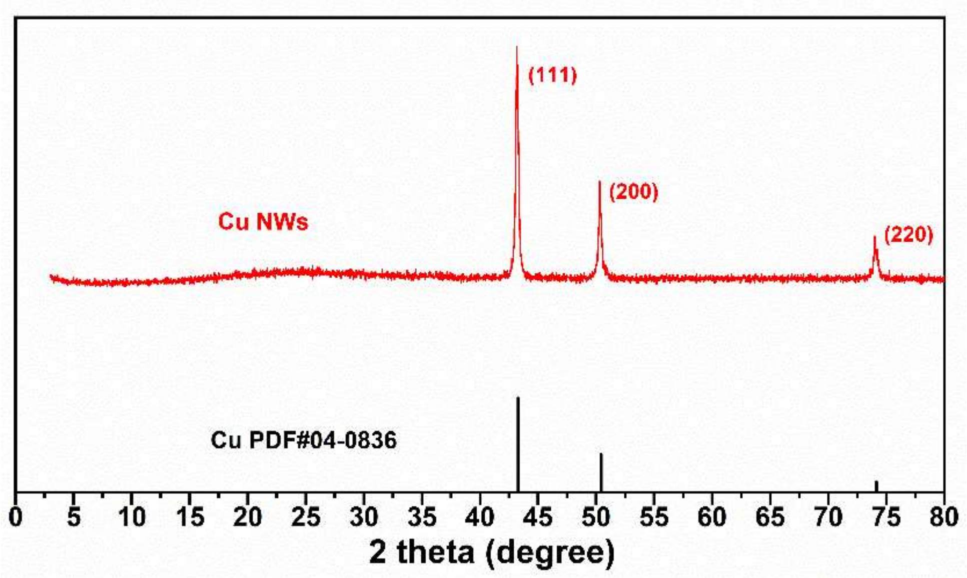

Figure S1. PXRD of Cu NWs.

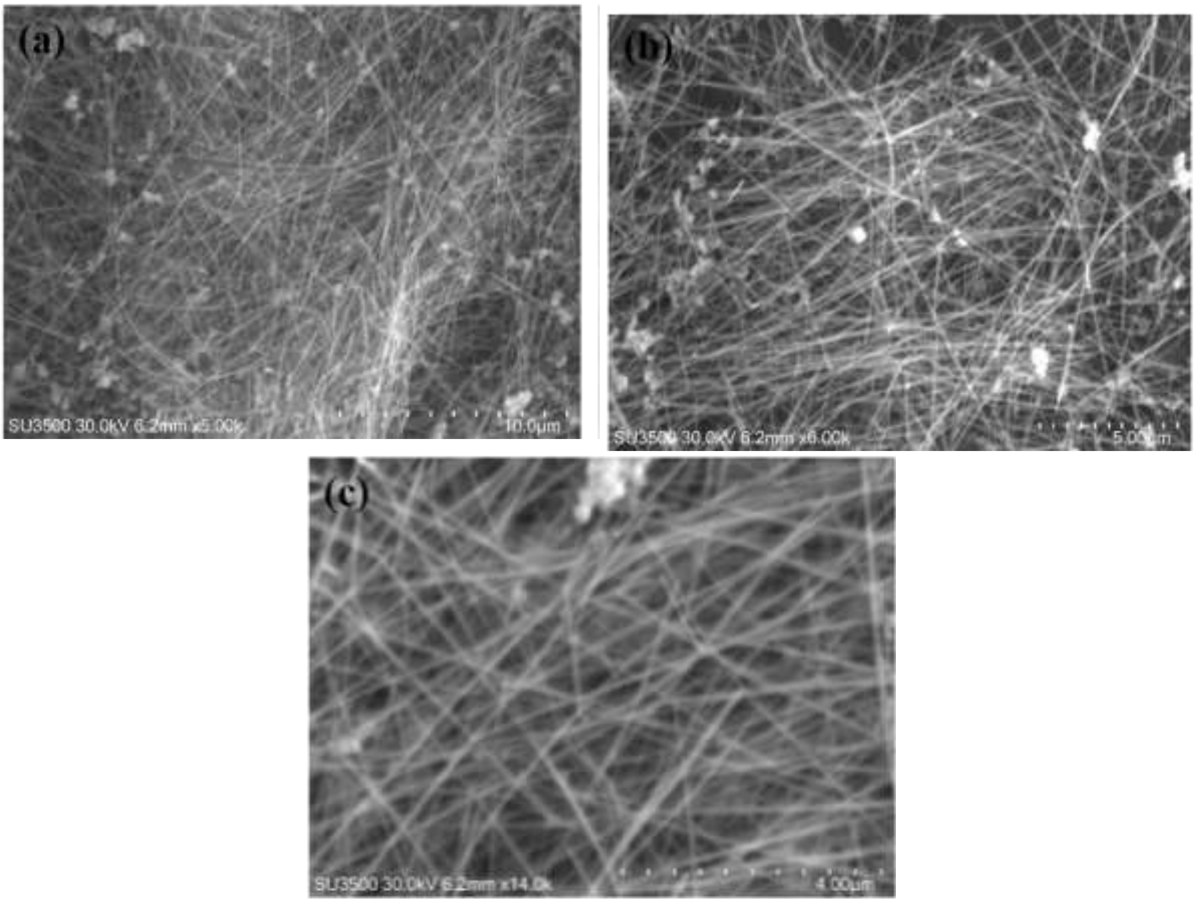

Figure S2. SEM of Cu NWs at different resolution. (scale bar: a, 10 μm; b, 5 μm; c, 4 μm).

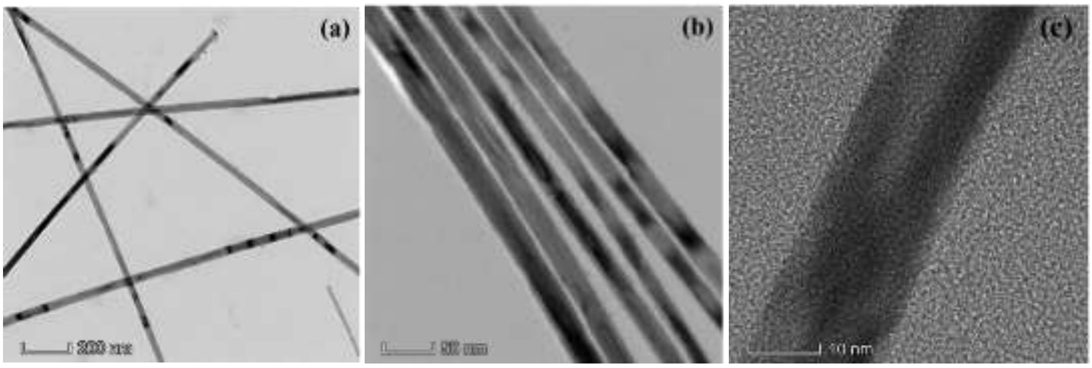

Figure S3. TEM of Cu NWs at different resolution. (scale bar: a, 200 nm; b, 50 nm; c, 10 nm).

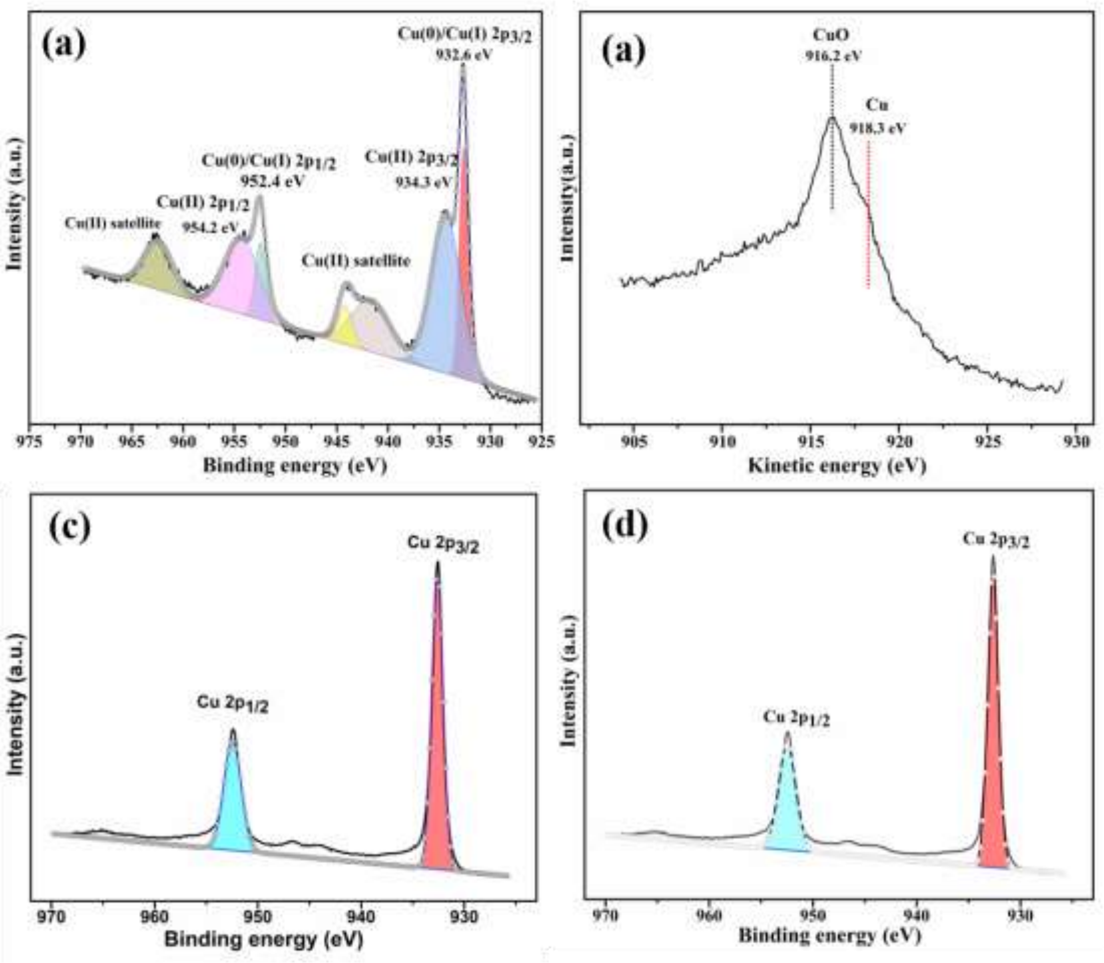

Figure S4. XPS of Cu NWs. (a), Cu 2p region of Cu NWs exposure to the ambient atmosphere. (b), Auger electron spectrum corresponding to sample a. (c), Cu 2p after surface etching 2 nm depth. (d), XPS after 50 cycles of CV activation.

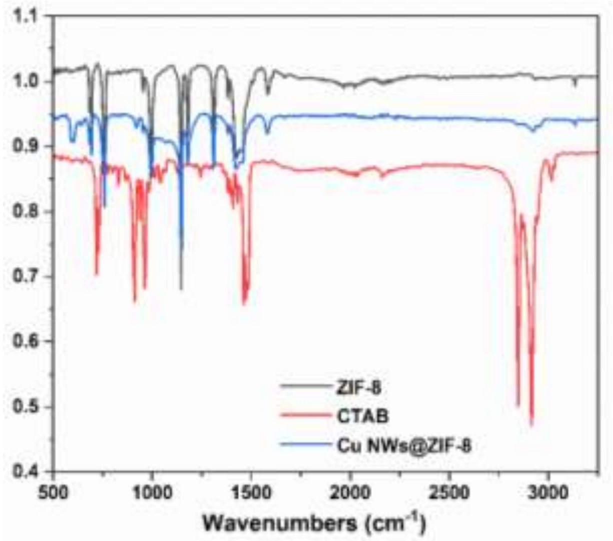

Figure S5. Infrared spectroscopy of Cu NWs@ZIF-8.

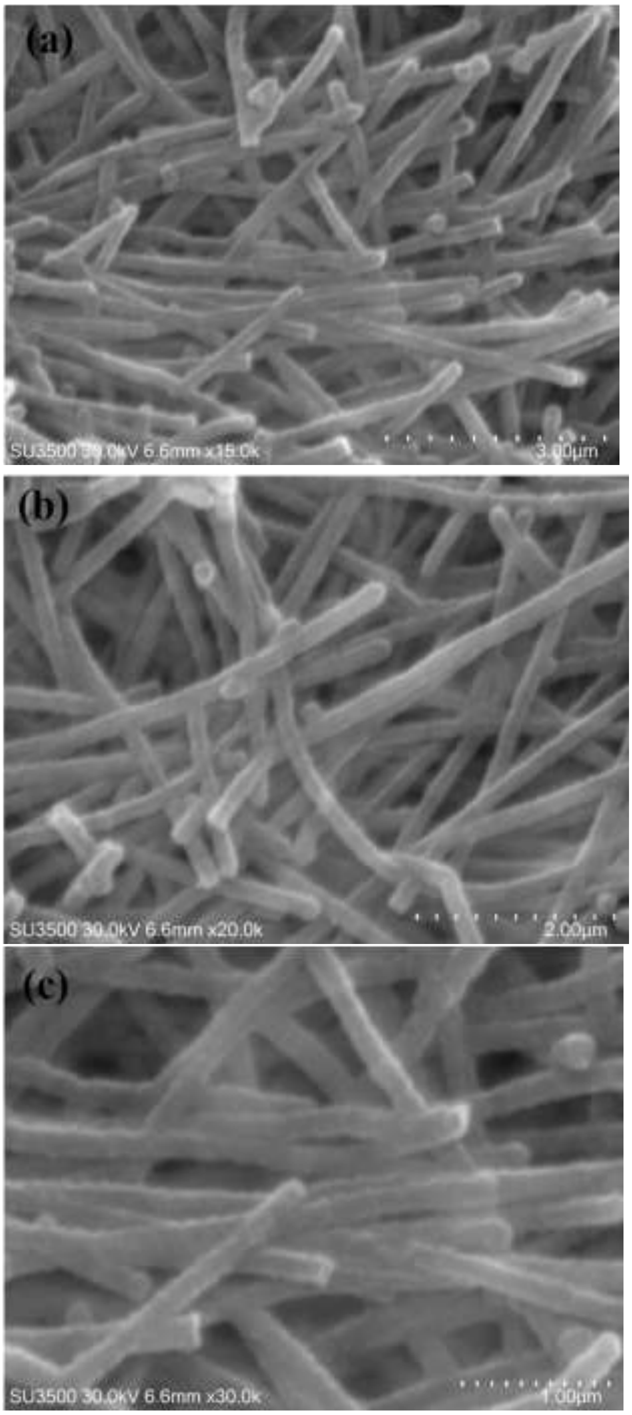

Figure S6. SEM images of Cu NWs@ZIF-8 with different resolution. (scale bar: a, 3 μm; b, 2 μm; c, 1 μm).

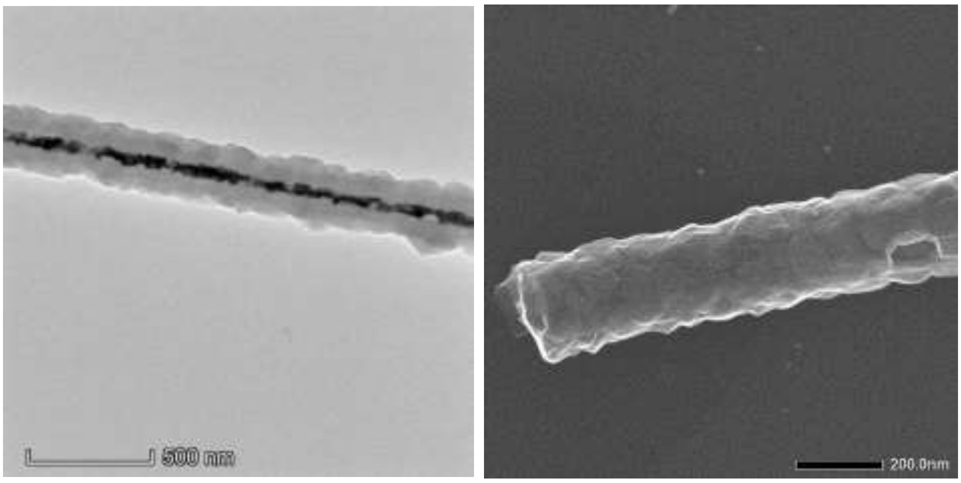

Figure S7. TEM and SEI images of Cu NWs@ZIF-8.

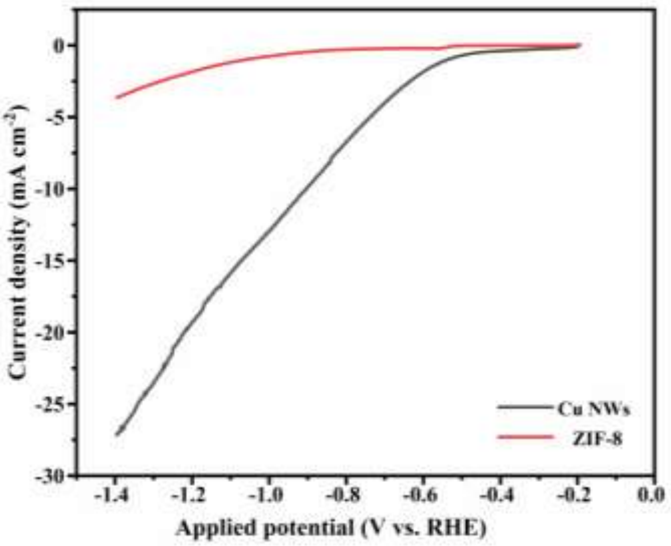

Figure S8. LSV of Cu NWs and ZIF-8 in CO_2 saturated 0.1 M $KHCO_3$ electrolyte.

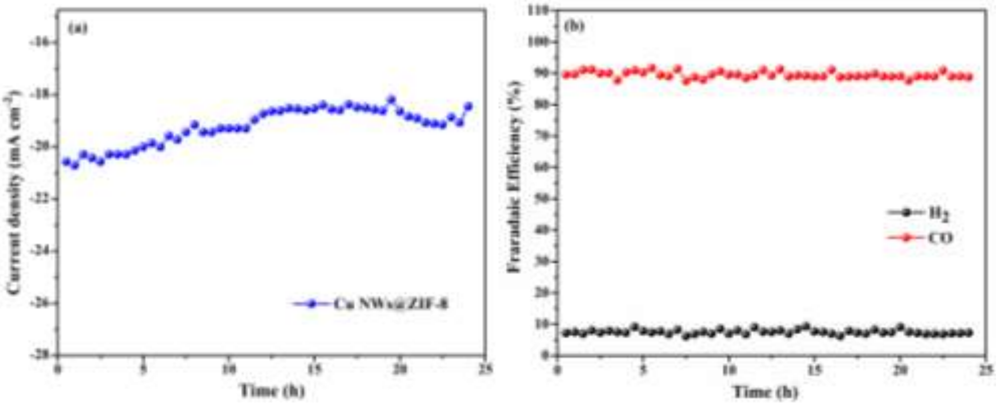

Figure S9. The long-term stability test for the Cu NWs@ZIF-8 catalyst. **a**, Total current density normalized to the geometric area of Cu NWs@ZIF-8 within 24 h. **b**, FE of CO and H_2 for Cu NWs@ZIF-8 within 24 h.

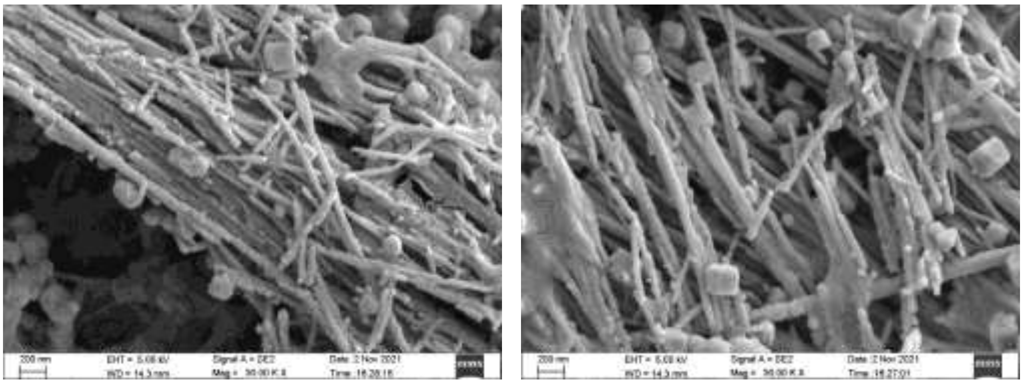

Figure S10: SEM images of Cu NWs@ZIF-8 after the reaction at -1.1 V_{RHE} for 2 h.

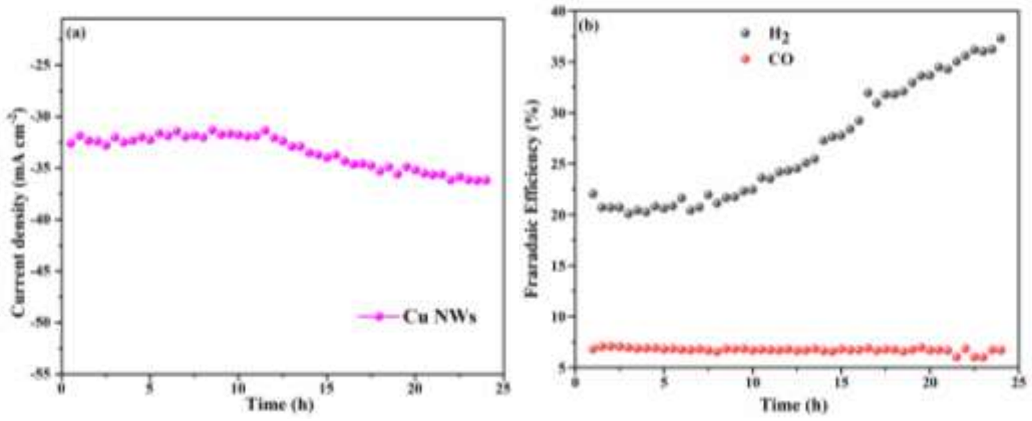

Figure S11. The long-term stability test for the Cu NWs. **a**, Total current density normalized to the geometric area of Cu NW within 24 h. **b**, FE of CO and H_2 for Cu NWs within 24 h.

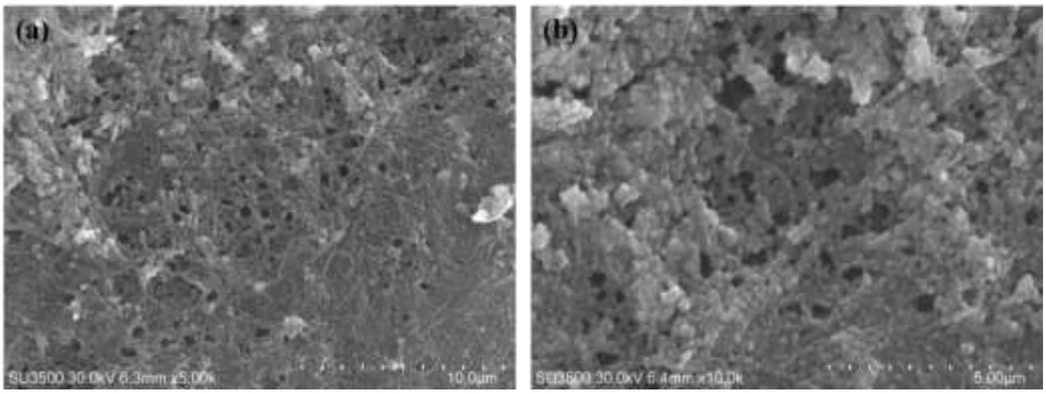

Figure S12. SEM images of Cu NWs after the reaction at -1.1 V_{RHE} for 2 h.

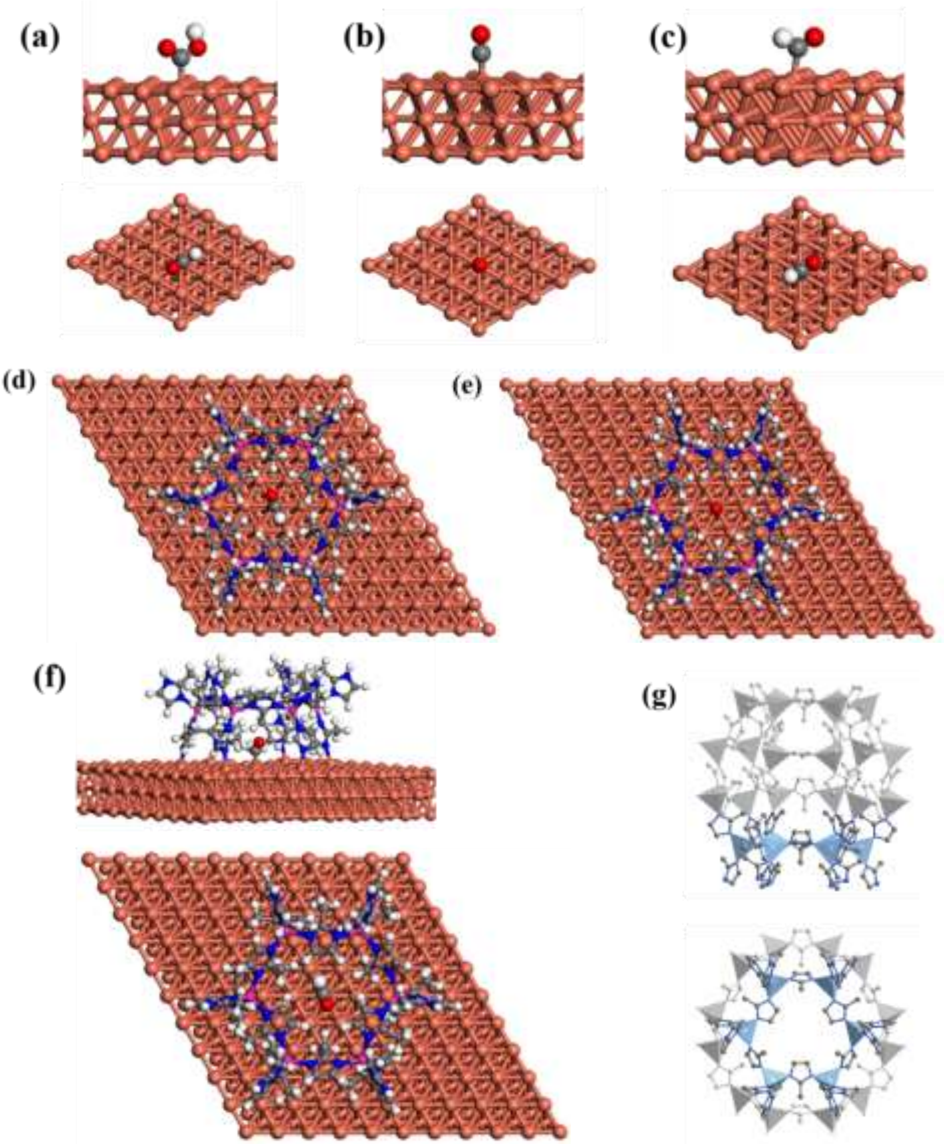

Figure S13. DFT optimized structure of intermediate *COOH, *CO and *CHO adsorbed on Cu(100) and Cu NWs@ZIF-8. (a) *COOH adsorbed on Cu(100); (b) *CO adsorbed on Cu(100); (c) *CHO adsorbed on Cu(100); (d) *COOH adsorbed on Cu NWs@ZIF-8; (e) *CO adsorbed on Cu NWs@ZIF-8; (f) *CHO adsorbed on Cu NWs@ZIF-8; (g) The structure of the ZIF-8 cage and the highlighted part is a window of the ZIF-8 cage. The gray, blue and light blue spheres represent C, N and Zn atoms, respectively.

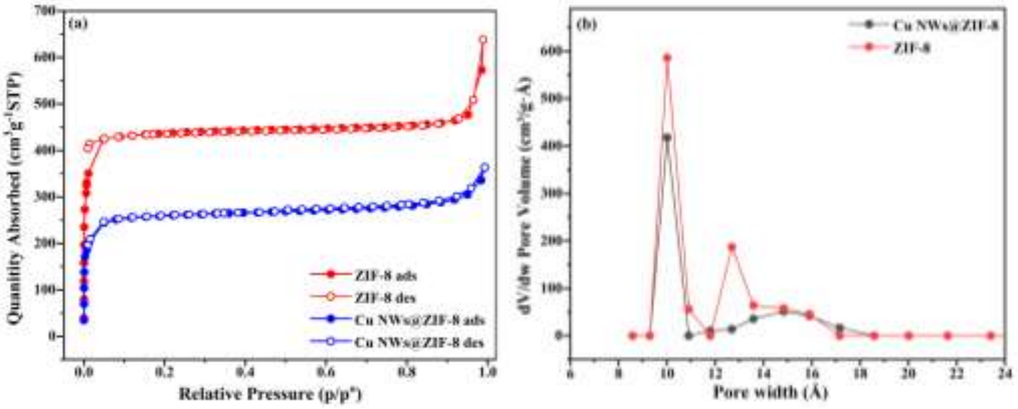

Figure S14. **a**, N_2 adsorption/desorption curves of ZIF-8 and Cu NWs@ZIF-8 at 77 K. **b**, Pore size distribution of ZIF-8 and Cu NWs@ZIF-8.

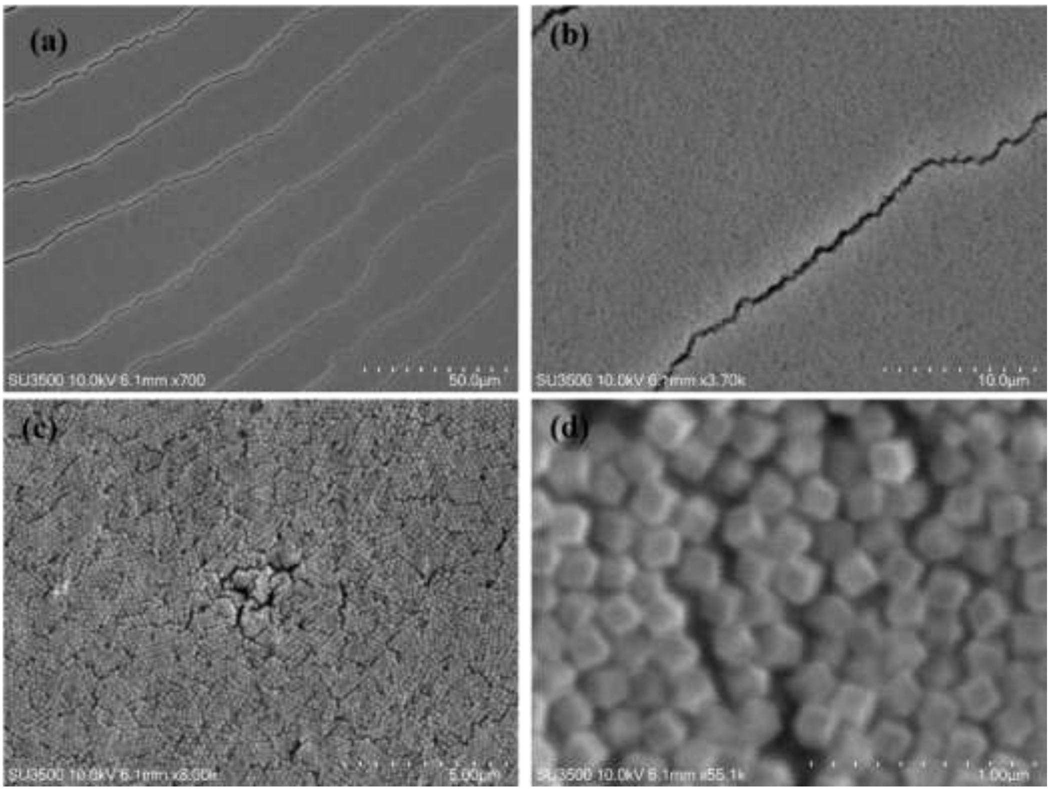

Figure S15. SEM images of ZIF-8 with different resolution. (scale bar: a, 50 μm; b, 10 μm; c, 5 μm; d, 1 μm).

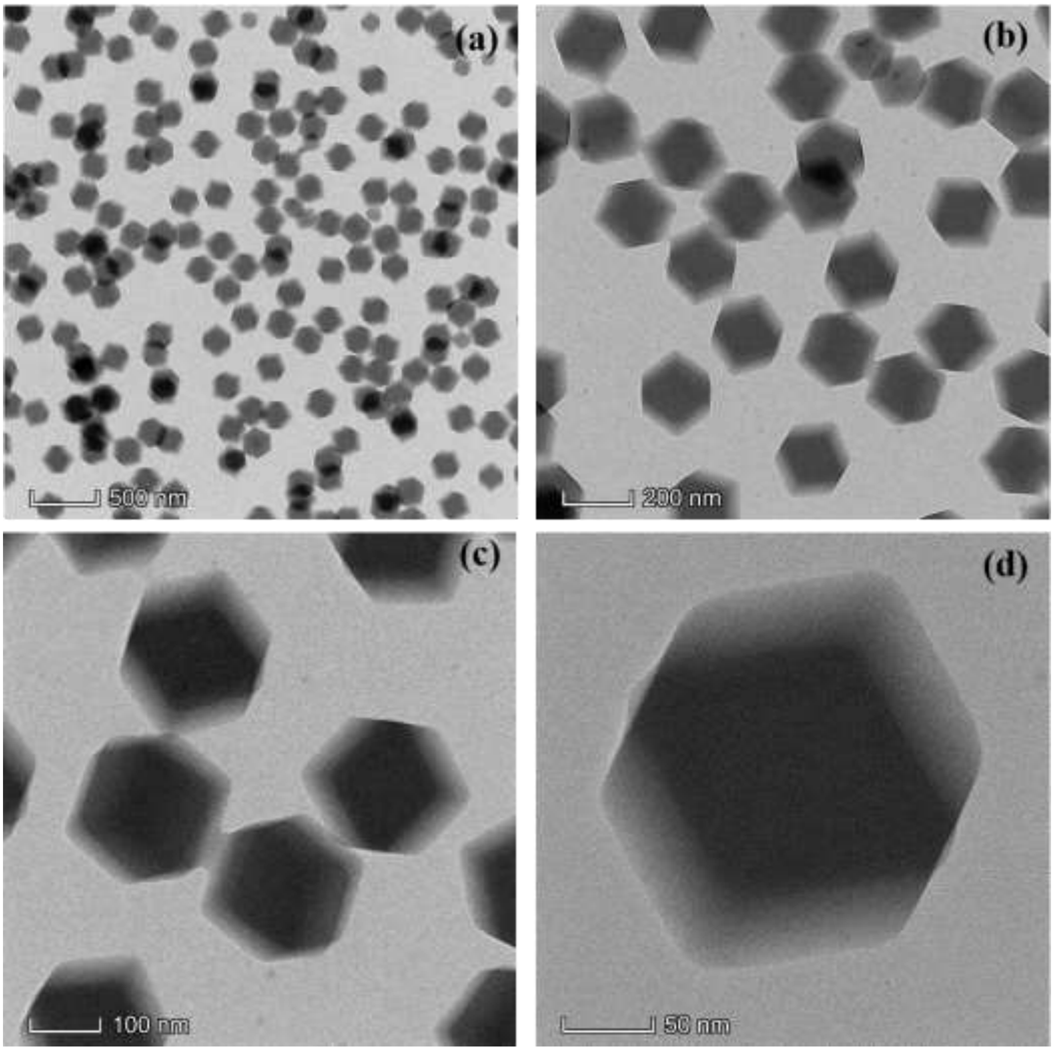

Figure S16. TEM images of ZIF-8 with different resolution. (scale bar: a, 500 nm; b, 200 nm; c, 100 nm; d, 50 nm)

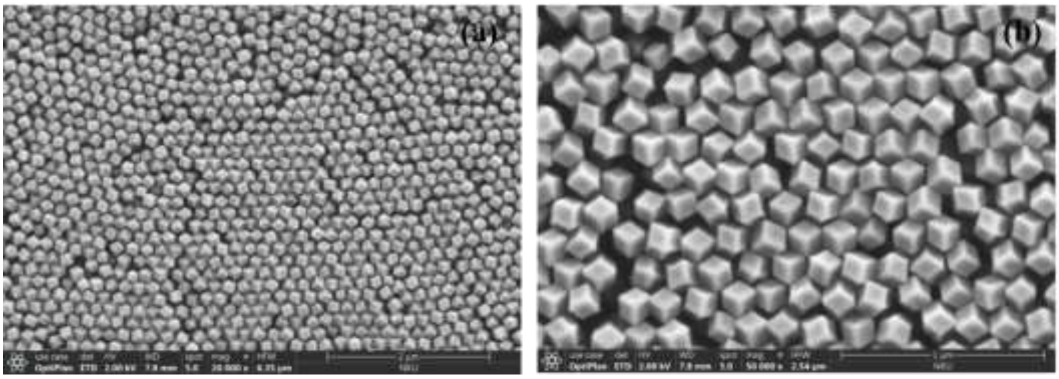

Figure S17. SEM images of ZIF-67 with different resolution. (scale bar: a, 2 μm; b, 1 μm)

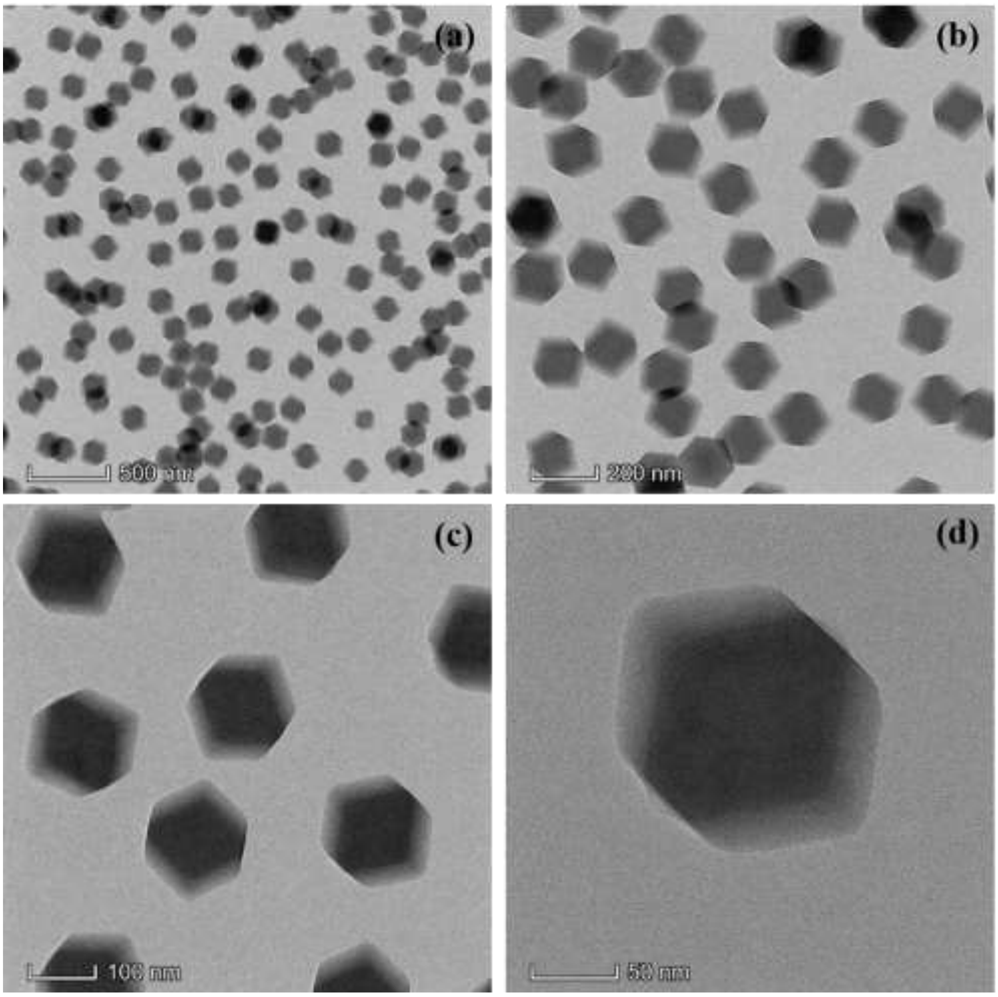

Figure S18. TEM images of ZIF-67 with different resolution. (scale bar: a, 500 nm; b, 200 nm; c, 100 nm; d, 50 nm)

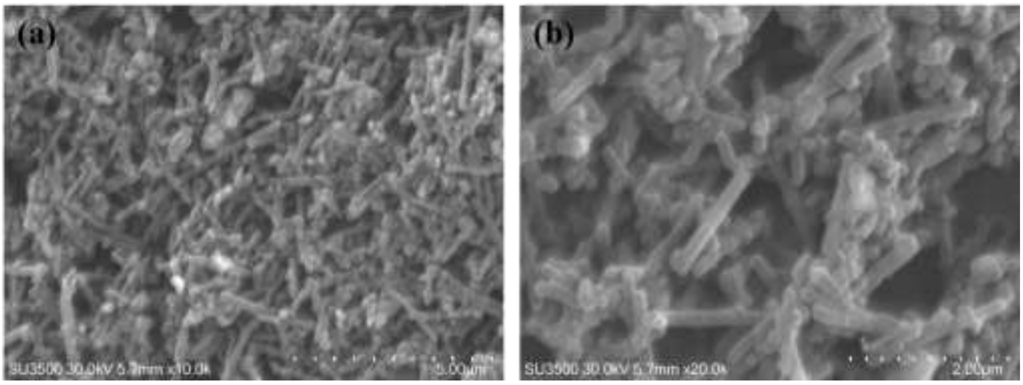

Figure S19. SEM images of Cu NWs@ZIF-67 with different resolution. (scale bar: a, 5 μm; b, 2 μm)

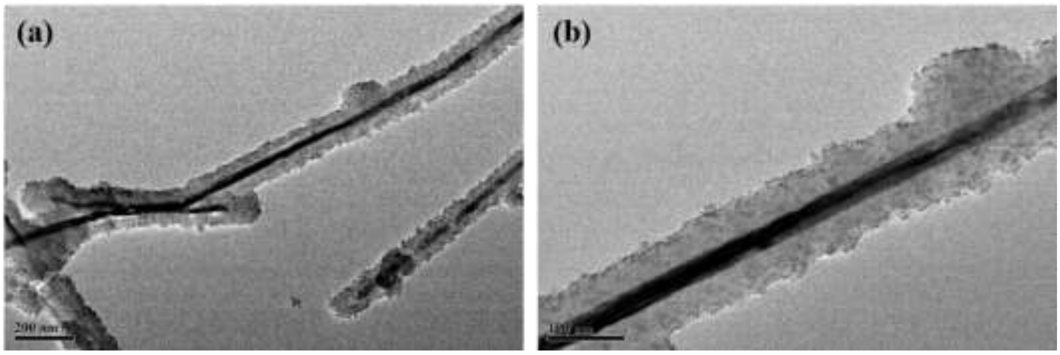

Figure S20. TEM images of Cu NWs@ZIF-67 with different resolution. (scale bar: a, 200 nm; b, 100 nm).

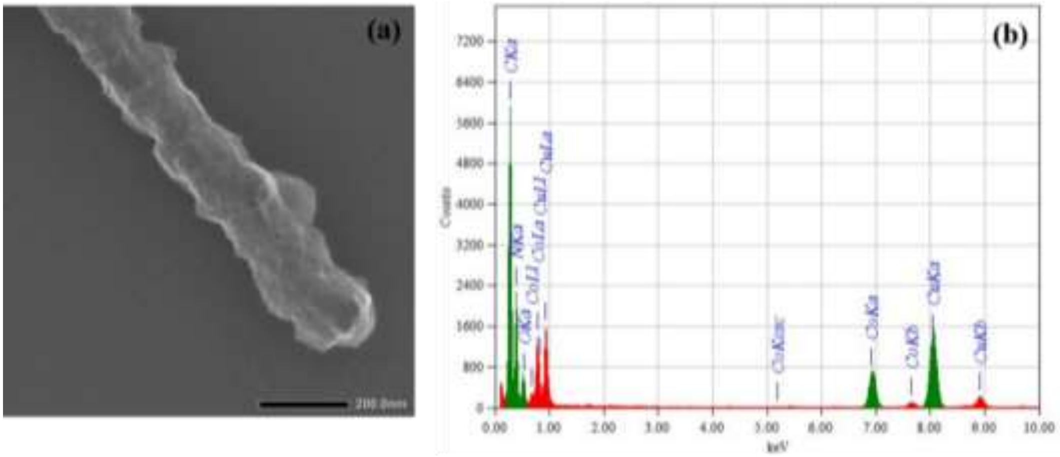

Figure S21. SEI image (a) and EDX image (b) of Cu NWs@ZIF-67.

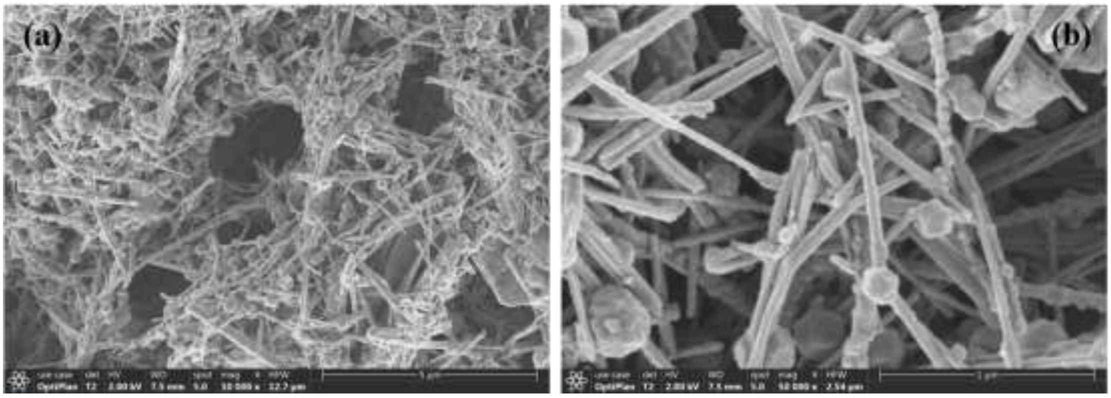

Figure S22. SEM images of Cu NWs@$Zn_{0.5}Co_{0.5}$-ZIF with different resolution. (scale bar: a, 5 μm; b, 1 μm).

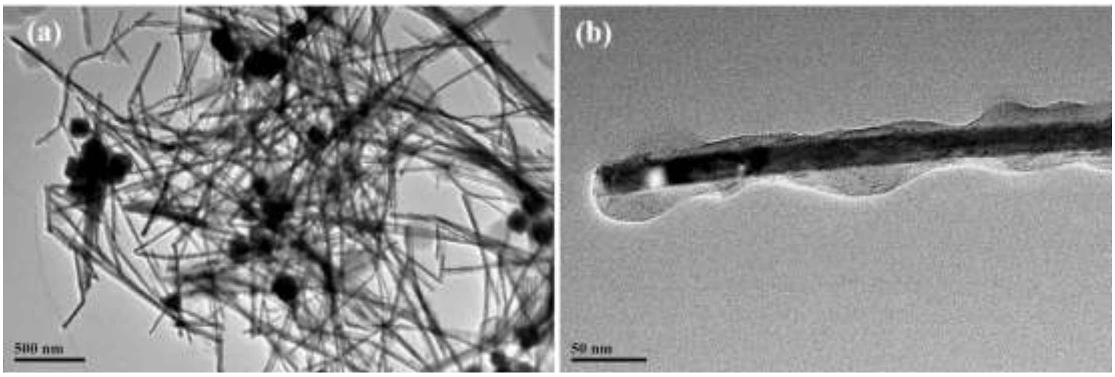

Figure S23. TEM images of Cu NWs@$Zn_{0.5}Co_{0.5}$-ZIF with different resolution. (scale bar: a, 500 nm; b, 50 nm).

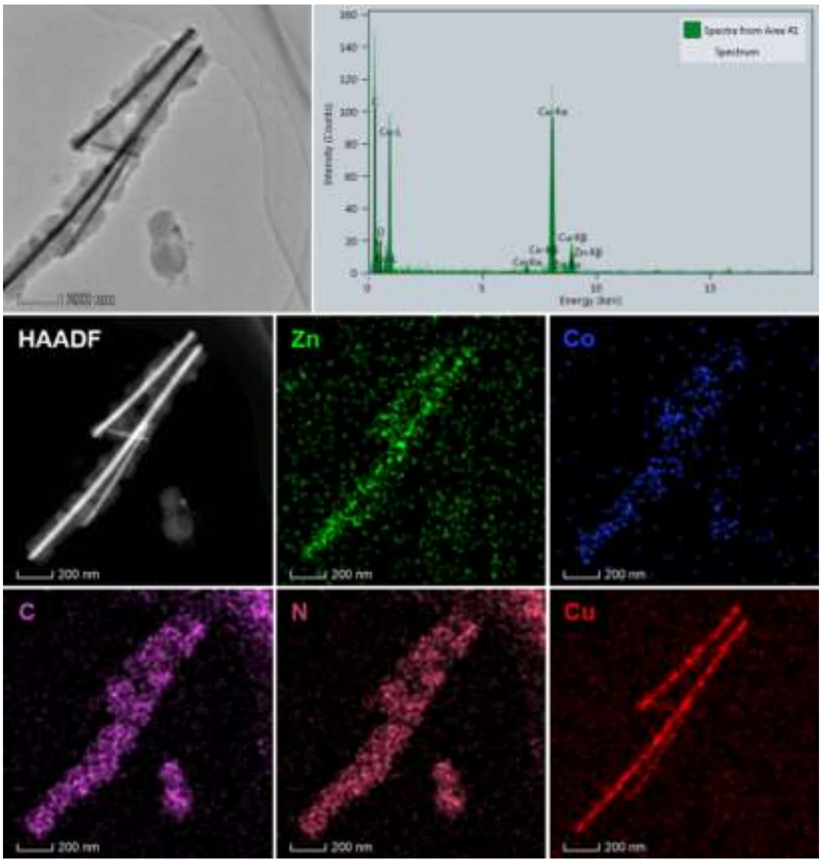

Figure S24. HAADF-STEM elemental maps (Zn, Co, C, N, Cu) and EDX image of Cu NWs@$Zn_{0.5}Co_{0.5}$-ZIF.

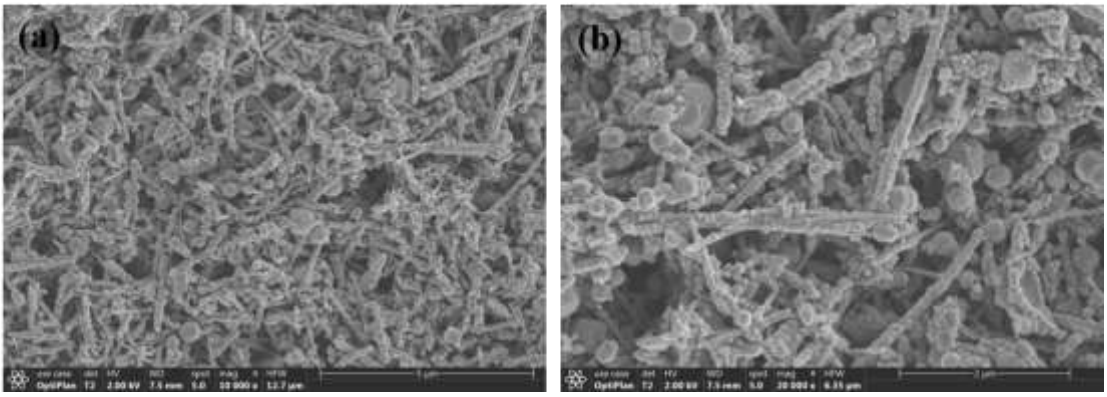

Figure S25. SEM images of Cu NWs@$Zn_{0.4}Co_{0.6}$-ZIF with different resolution. (scale bar: a, 5 μm; b, 2 μm).

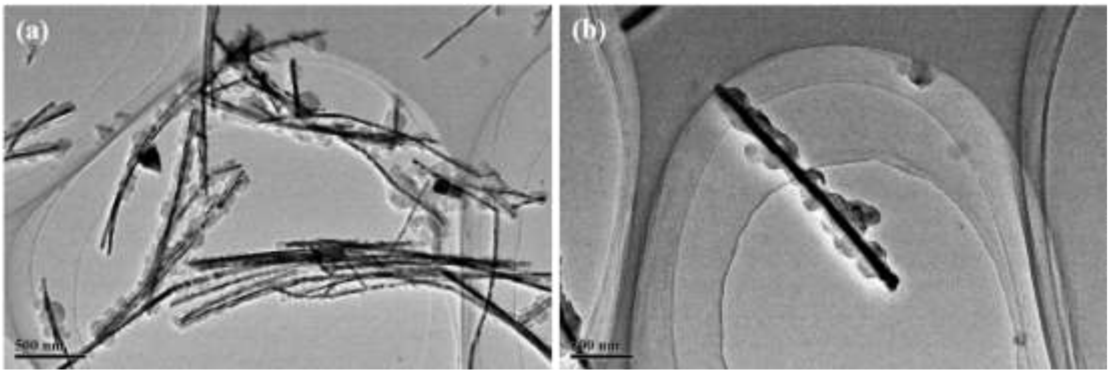

Figure S26. TEM images of Cu NWs@$Zn_{0.4}Co_{0.6}$-ZIF with different resolution. (scale bar: a, 500 nm; b, 200 nm).

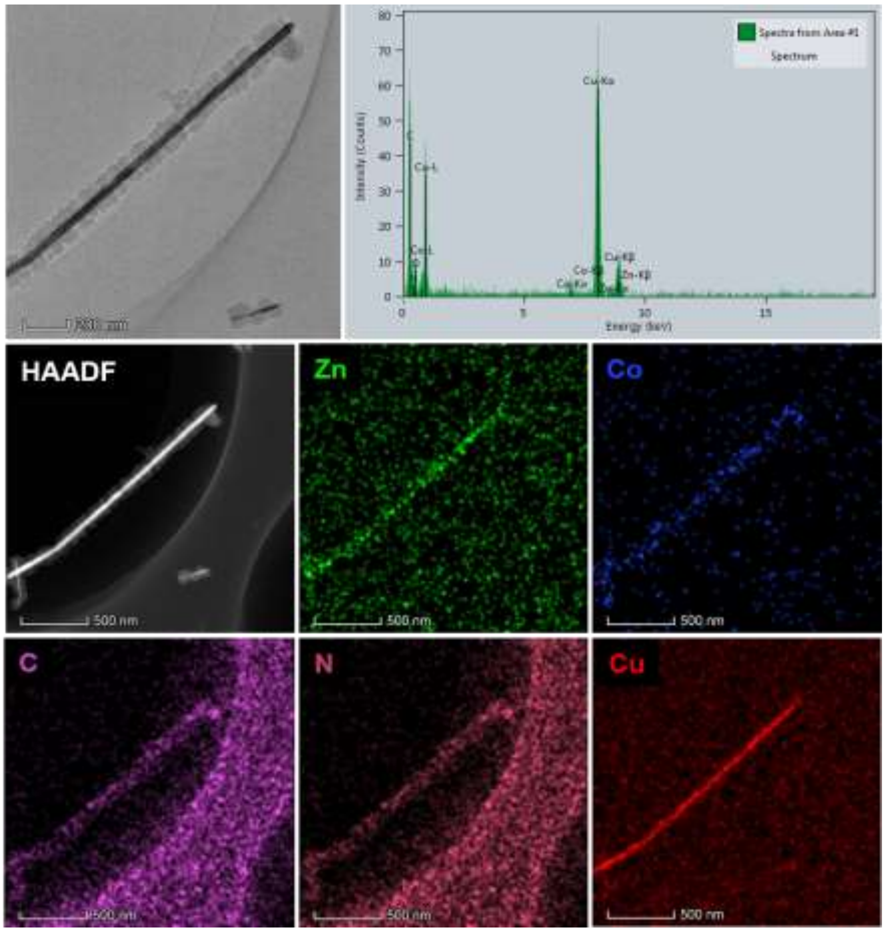

Figure S27. HAADF-STEM elemental maps (Zn, Co, C, N, Cu) and EDX image of Cu NWs@$Zn_{0.4}Co_{0.6}$-ZIF.

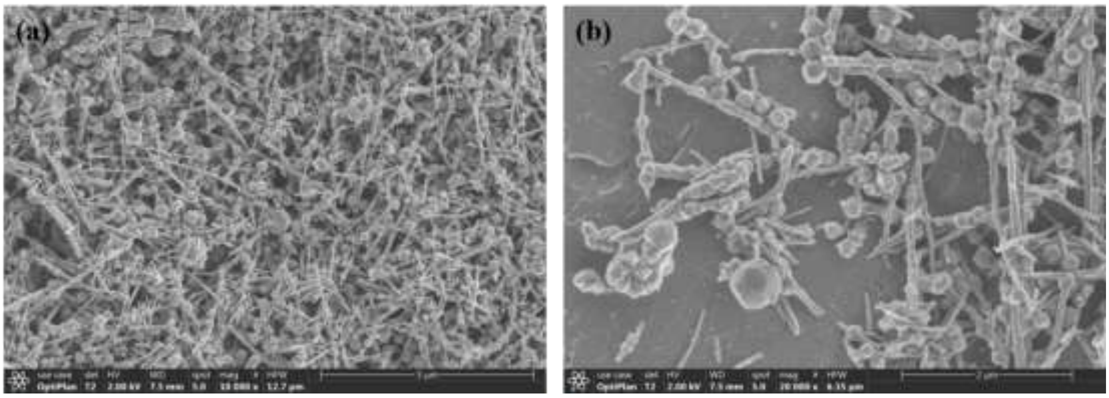

Figure S28. SEM images of Cu NWs@$Zn_{0.3}Co_{0.7}$-ZIF with different resolution. (scale bar: a, 5 μm; b, 2 μm).

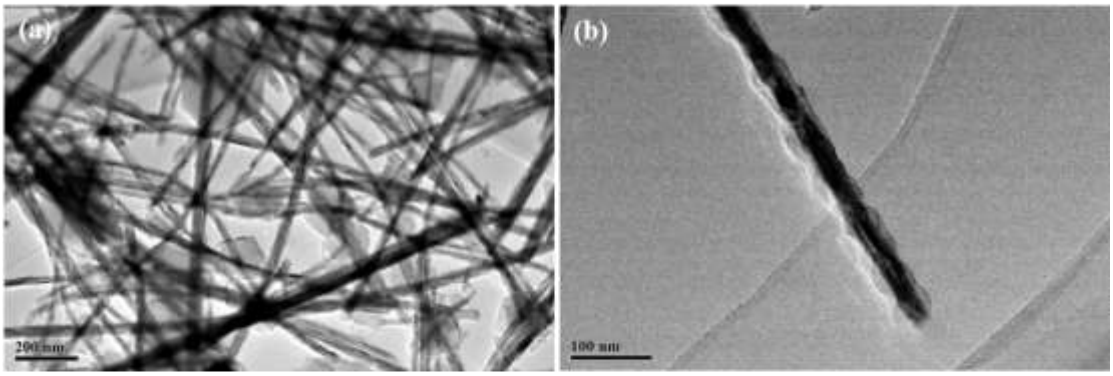

Figure S29. TEM images of Cu NWs@$Zn_{0.3}Co_{0.7}$-ZIF with different resolution. (scale bar: a, 200 nm; b, 100 nm).

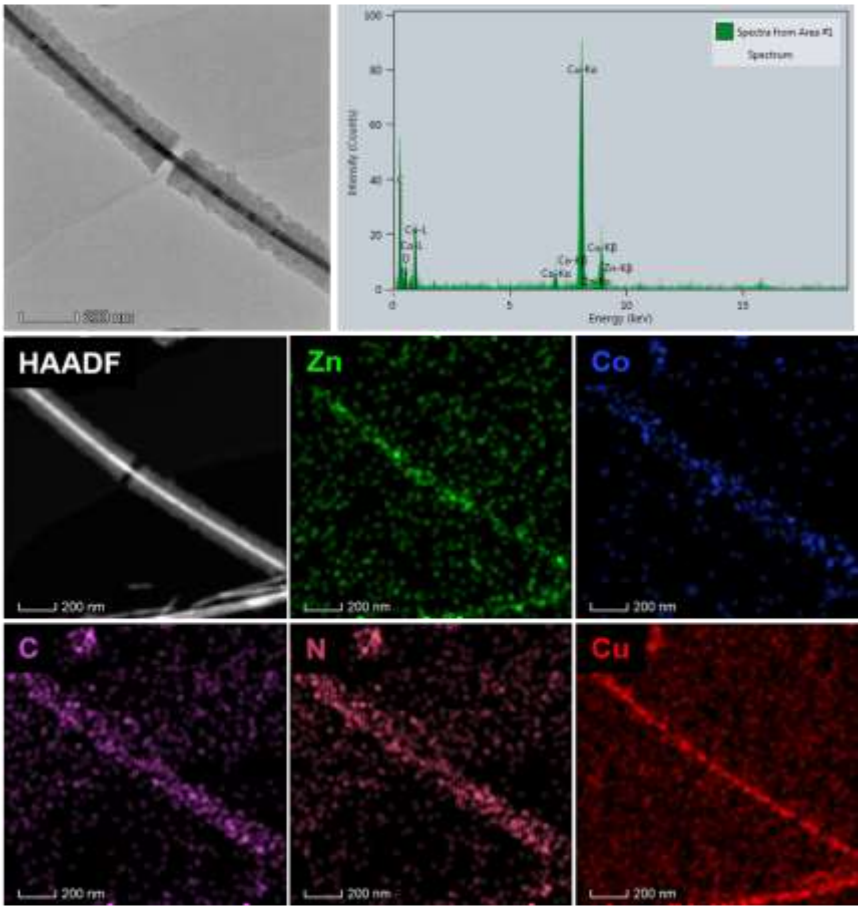

Figure S30. HAADF-STEM elemental maps (Zn, Co, C, N, Cu) and EDX image of Cu NWs@$Zn_{0.3}Co_{0.7}$-ZIF.

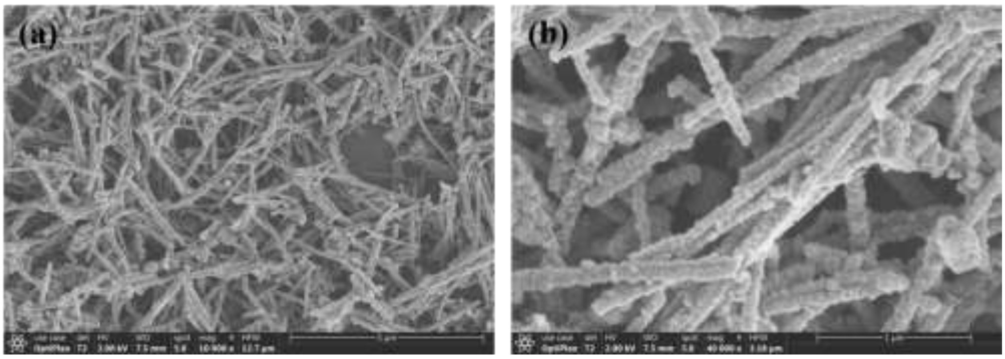

Figure S31. SEM images of Cu NWs@$Zn_{0.2}Co_{0.8}$-ZIF with different resolution. (scale bar: a, 5 μm; b, 1 μm).

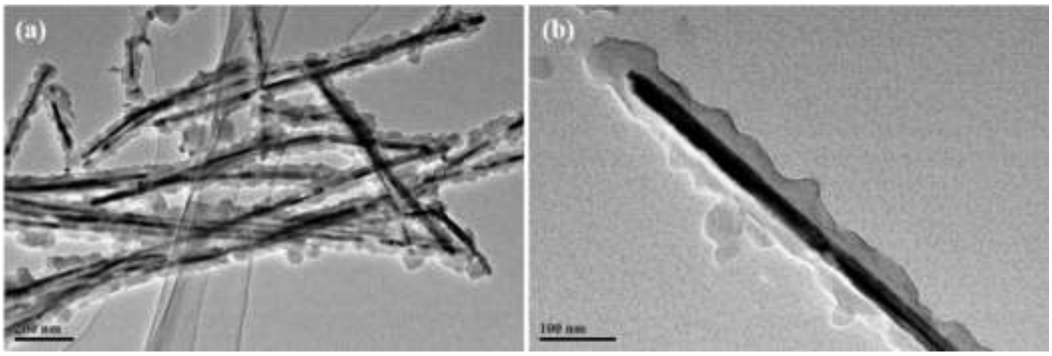

Figure S32. TEM images of Cu NWs@$Zn_{0.2}Co_{0.8}$-ZIF with different resolution. (scale bar: a, 200 nm; b, 100 nm).

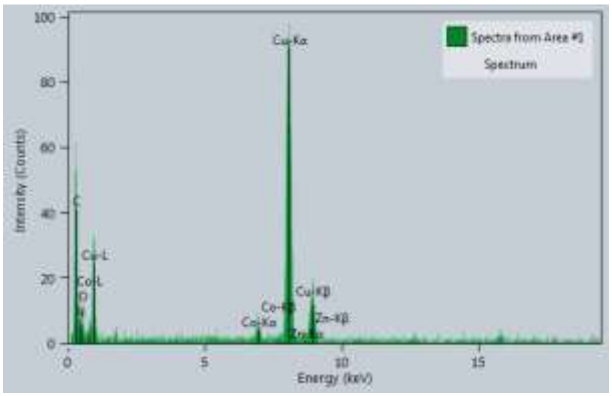

Figure S33. EDX image of Cu NWs@$Zn_{0.2}Co_{0.8}$-ZIF.

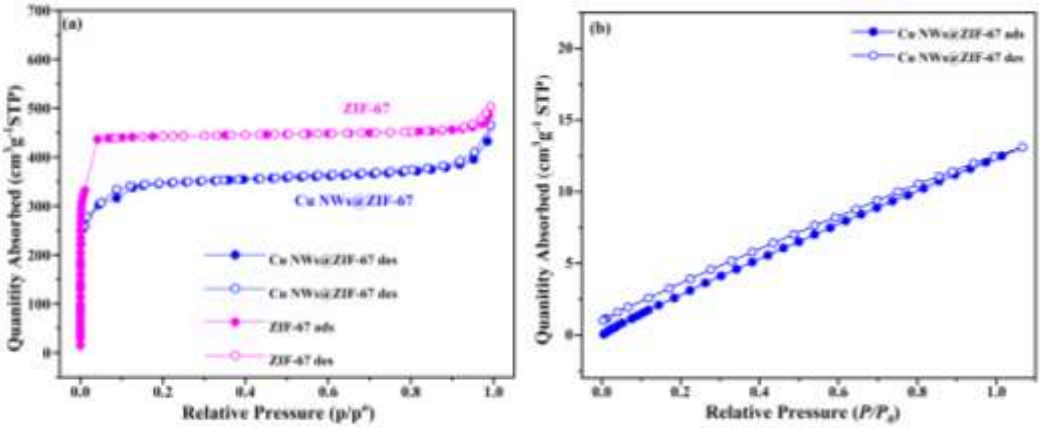

Figure S34. N_2 adsorption/desorption curves (a) at 77K and CO_2 uptake amount (b) at 298K of ZIF-67 and Cu NWs@ZIF-67.

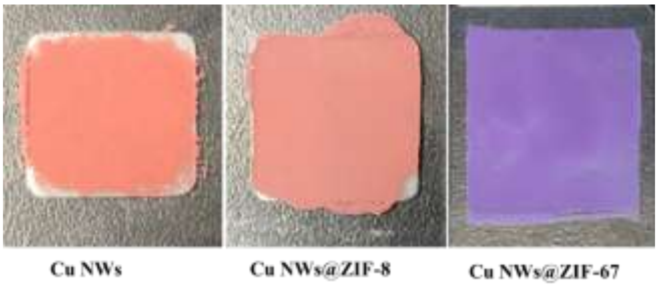

Figure S35. Optical photos of Cu NWs, Cu NWs@ZIF-8 and Cu NWs@ZIF-67.

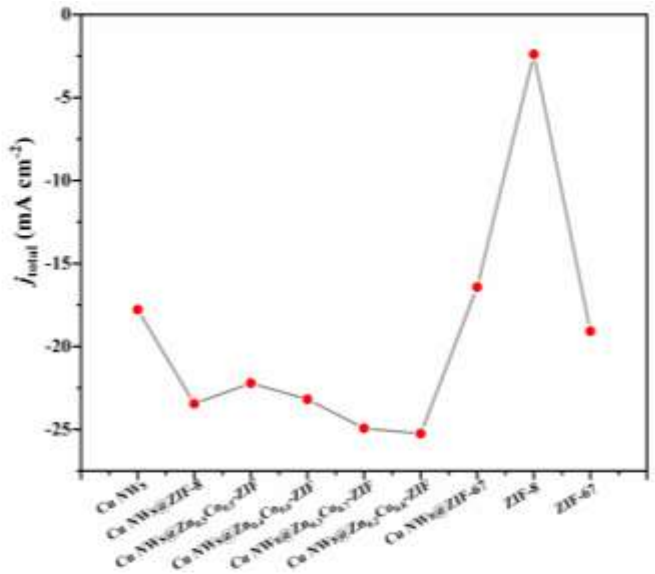

Figure S36. Total current densities for the different types of catalysts in 0.1 M $KHCO_3$ at -1.1 V_{RHE}. The current densities were normalized to the geometric area.

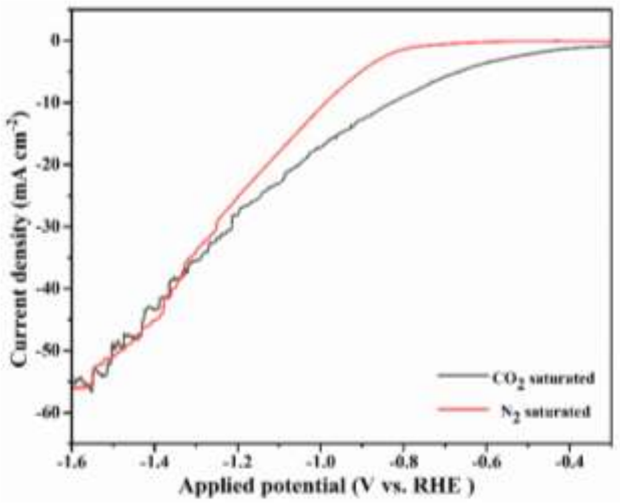

Figure S37. LSV of ZIF-67 in N_2 and CO_2 saturated 0.1 M $KHCO_3$ electrolyte.

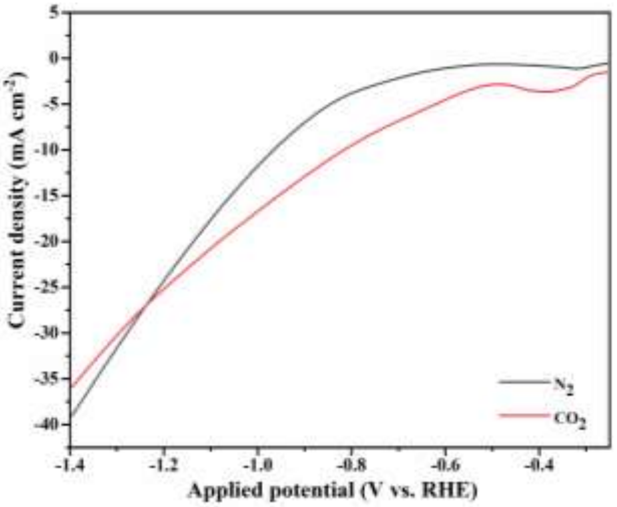

Figure S38. LSV of Cu NWs@ZIF-67 in N_2 and CO_2 saturated 0.1 M $KHCO_3$ electrolyte.

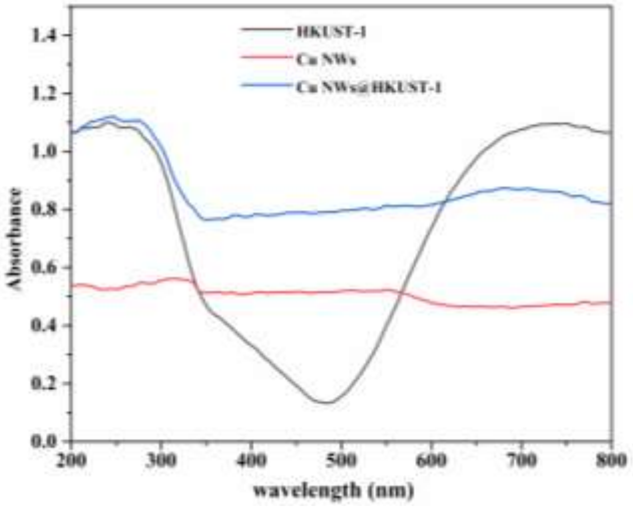

Figure S39. UV-visible diffuse reflection spectra for Cu NWs, HKUST-1 and Cu NWs@HKUST-1.

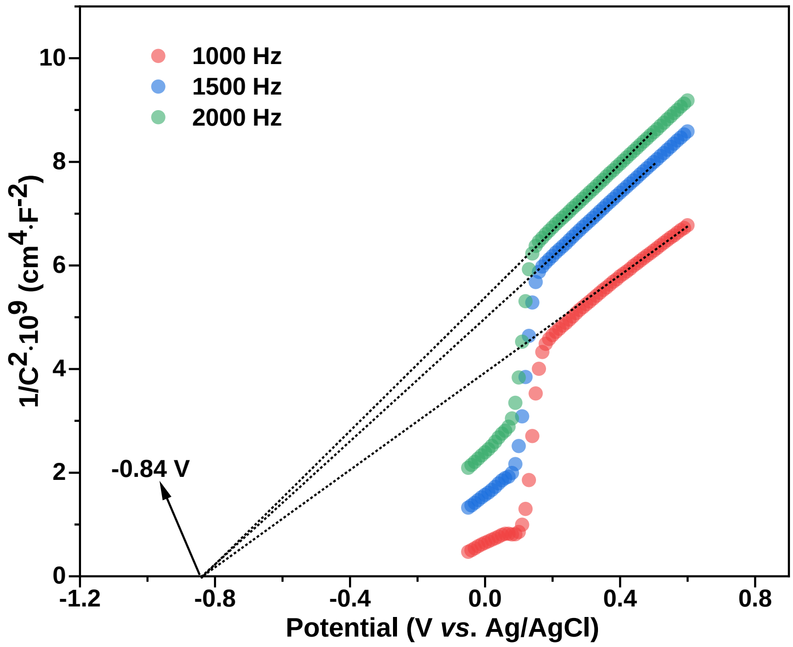

Figure S40. Mott-Schottky plots of HKUST-1.

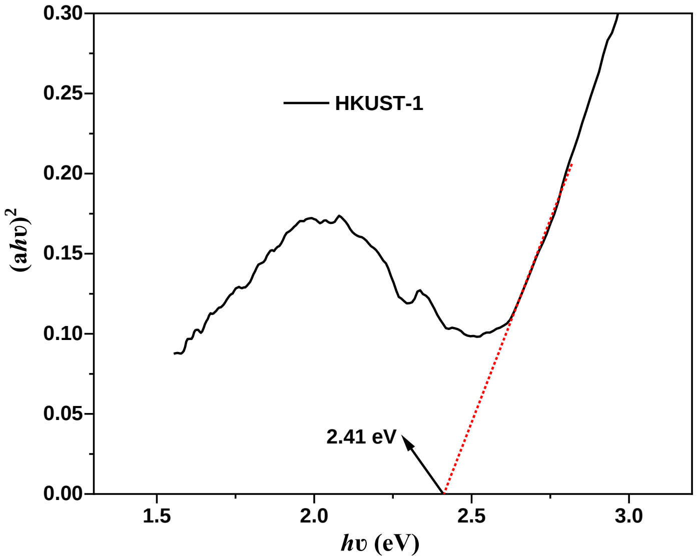

Figure S41. Tauc plots of HKUST-1.

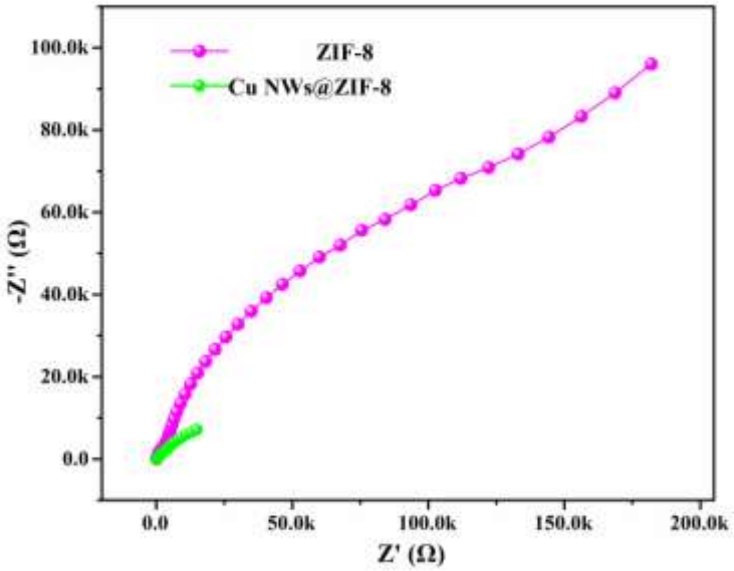

Figure S42. The comparison of the Nyquist polt of ZIF-8 and Cu NWs@ZIF-8 catalysts conducted at 0 V vs. open-circuit potential from 0.01 Hz to 100 kHz with amplitude of 5 mV.

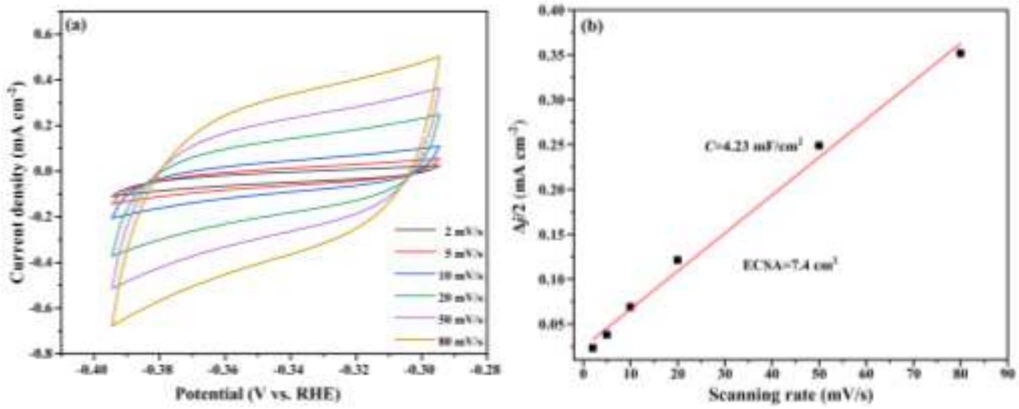

Figure S43. Double-layer capacitance values and (b) for Cu NWs@ZIF-8 according to cyclic voltammetry (CV) measurements (a).

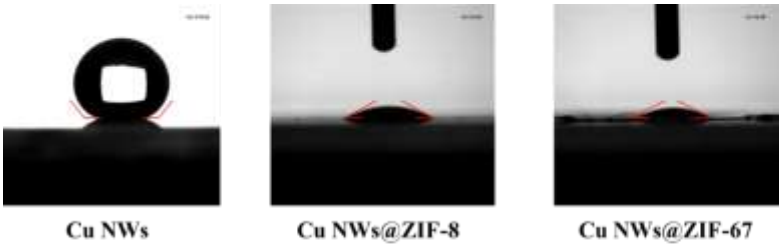

Figure S44. Contact angles tests of Cu NWs, Cu NWs@ZIF-8 and Cu NWs@ZIF-67 to water.

Table S1. Physical and chemical parameters of ZIF-8, ZIF-67, Cu NWs@ZIF-8 and Cu NWs@ZIF-67.

Sample	BET surface area (m2/g)	Content of Cu (wt %)
ZIF-8	1291.24	/
ZIF-67	1459.46	/
Cu NWs@ZIF-8	800.15	22.2
Cu NWs@ZIF-67	1044.64	20.7

Table S2. Recent advances of Cu-based catalysts for electrocatalytic reduction CO_2 to CO/H_2.

Catalysts	electrolyte	V vs. RHE	cell	FE/j	Products	stability	ref.
Cu NWs@ZIF-8	0.1 M $KHCO_3$	-1.1	H-cell	91.3	CO	24 h	This work
Cu NCs	0.5 M $KHCO_3$	-0.65	H-cell	20 mA cm^{-2}	CO/H2	8 h	*J. Am. Chem. Soc*. 2017, 139, 9359
Cubic Cu_2O	0.1 M $KHCO_3$	-0.86	H-cell	18	CO	5 h	*Angew. Chem. Int. Ed.* 2020,59, 17974-17983
Cu_2O@ZIF-8	0.1 M $KHCO_3$	-0.7~-1.1	H-cell	90.3	CO/H_2	12 h	*Angew. Chem. Int. Ed*. 2022, 61, e202116736
Pd/Cu	0.1 $KHCO_3$	-1.3	H-cell	6 mA cm^{-2}	CO/H_2	10 h	*Chem. Mater.* 2022, 34, 17, 7995-8003
Cu_2O/ZnO	0.1 $KHCO_3$	-1.0	H-cell	65.7	CO/H_2	2 h	*J. CO2 Util*, 2023, 68, 102368
Cu/In_2O_3	0.1 $KHCO_3$	-0.9	H-cell	31.1	CO/H_2	/	*ChemSusChem, 2022,15, e2022013*
Ag–Cu	0.1 $KHCO_3$	-0.7	H-cell	5.6 mA cm^{-2}	CO	10 h	*Nano Energy*, 2020, 73, 104796
Cu–Zn	0.1 $KHCO_3$	-1.0	H-cell	94	CO	15 h	*ACS Catal.* 2022, 12, 2741-2748

Table S3. The metal content (Zn, Co) in the Cu NWs@Zn_xCo_{1-x}-ZIF composites obtained from STEM-EDS.

Sample	Zn (atom%)	Co (atom%)	Cu (atom%)
Cu NWs@$Zn_{0.5}Co_{0.5}$-ZIF	0.35	0.74	24.89
Cu NWs@$Zn_{0.4}Co_{0.6}$-ZIF	0.24	0.9	32.79
Cu NWs@$Zn_{0.3}Co_{0.7}$-ZIF	0.41	1.66	42.11
Cu NWs@$Zn_{0.2}Co_{0.8}$-ZIF	0.31	1.72	42.64
